# Corrosion inhibition of a novel antihistamine-based compound for mild steel in hydrochloric acid solution: experimental and computational studies

**DOI:** 10.1038/s41598-022-17589-y

**Published:** 2022-08-04

**Authors:** Mohammad Ghaderi, Ahmad Ramazani S. A., Azadeh Kordzadeh, Mohammad Mahdavian, Eiman Alibakhshi, Arash Ghaderi

**Affiliations:** 1 Department of Chemical and Petroleum Engineering, Sharif University of Technology, Tehran, Iran; 2 Surface Coating and Corrosion Department, Institute for Color Science and Technology, Tehran, Iran; 3 Atlas Protecting Coating Company, Tehran, Iran; 4 Department of Chemistry, College of Sciences, University of Hormozgan, Bandar Abbas, 7916193145 Iran

**Keywords:** Corrosion, Surface chemistry, Theoretical chemistry

## Abstract

Focused on the assessment of the diphenhydramine hydrochloride (DPH) capabilities as an alternative to conventional and harmful industrial corrosion inhibitors, electrochemical techniques were employed. The optimum concentration of 1000 ppm was determined by molecular simulation and validated through electrochemical experiments. The results acquired from the electrochemical impedance spectroscopy (EIS) study showed that DPH at a concentration of 1000 ppm has a corrosion efficiency of 91.43% after 6 h immersion. The DPH molecules' orientation on the surface was assessed based on EIS predicting horizontal adsorption on the surface. Molecular simulations were done to explore the adsorption mechanism of DPH. The DPH molecules' orientation on the surface was also assessed based on computational studies confirming the horizontal adsorption predicted by EIS.

## Introduction

The distinctive qualities of mild steel (MS), such as good mechanical properties and low density, make it an indispensable popular choice for usage in various sectors, including oil refineries, desalination plants, automotive, shipbuilding, and construction^1^. Pickling, acidizing, descaling, and other procedures are used to remove rust and iron oxide from the steel surface. Hydrochloric acid (HCl) is one of the most commonly utilized solutions for this purpose. Unfortunately, MS is corroded by exposure to HCl, and as a result, its mechanical properties are reduced and cause financial and human losses^2^.

Corrosion inhibitors are the most efficient compounds to reduce corrosion rates. Specifically, compounds containing heteroatoms such as nitrogen, oxygen, sulfur, and phosphorous, as well as those having π orbitals and conjugated multiple bonds, can be adsorbed on metal surfaces avoiding corrosion by producing a protective layer. There are three methods for adsorption of corrosion inhibitors on the metal surface: (1) Physical adsorption, which functions through the interaction of opposite charges of the metal surface and the inhibitor molecules, (2) Chemical adsorption, which functions by sharing the lone electron pair of the inhibitor molecules with the vacant d orbital of iron ions and (3) Physicochemical adsorption which is a combination of above methods^3–6^. Application of toxic corrosion inhibitors has been restricted^7^; consequently, researchers have turned to environmentally friendly inhibitors such as plant extracts^8–13^, biopolymers^14,15^, and ionic liquids^16–18^ in their attempts to mitigate corrosion damages. Expired drugs are another class of substances that have the potential to be used as corrosion inhibitors. These compounds are usually composed of aromatic rings and heteroatoms, and they are generally considered to be environmentally friendly. For this reason, they are expected to have excellent inhibition properties. Qiang et al. used Losartan potassium as a corrosion inhibitor for MS in 1 M HCl solution and achieved an efficiency of 88.9% at a concentration of 5 mM at room temperature^19^. Kumar et al. studied the effect of cefazolin on decreasing steel corrosion at temperatures ranging from 308 to 338 K. The results indicated that this mixed-type inhibitor followed the Langmuir isotherm. In addition, the corrosion rate decreased as corrosion inhibitor concentration increased^20^. Bashir et al. studied Analgin's corrosion-inhibiting capabilities using electrochemical impedance spectroscopy (EIS), potentiodynamic polarization (PDP), and weight loss methods^21^. Other drugs have also been used to prevent corrosion in acidic environments including Sulfa drugs^22^, Pheniramine drugs^23^, Streptomycin^24^, Metformin^25^, Pencilin G, Ampicillin, Amoxicillin^26^, Cefalexin^27^, Ketosulfone^28^, Cefixime^29^, Atorvastatin^30^, Septazole^31^, Pantoprazole sodium^32^ and Ondansetron^33^.

**Figure 1:**
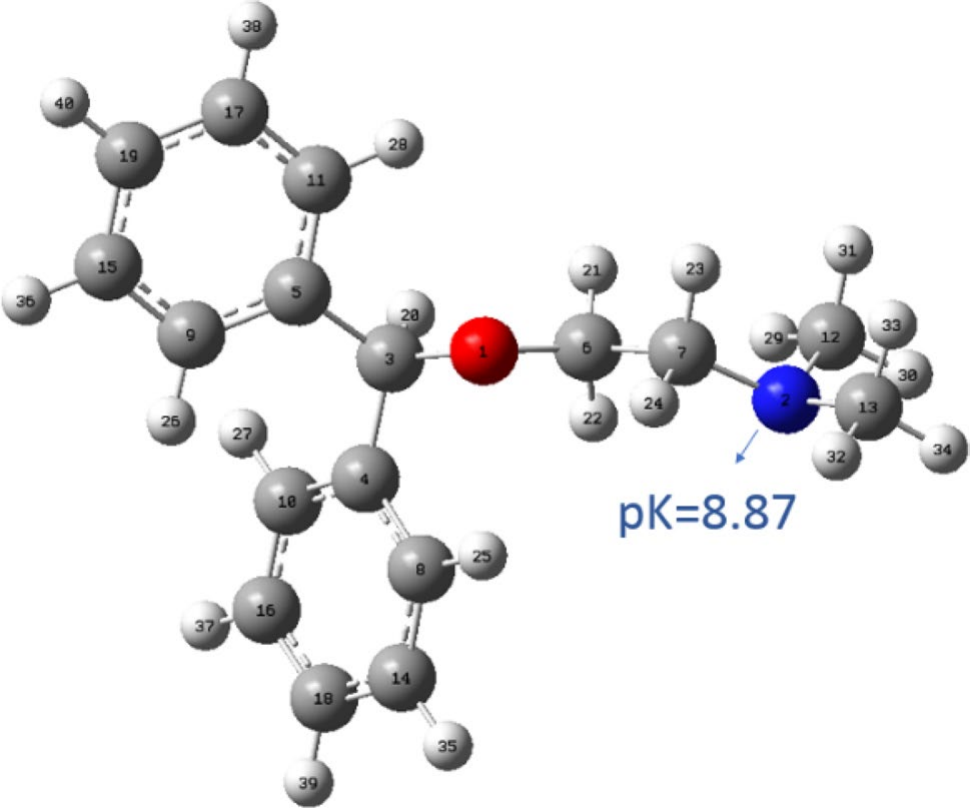
The chemical structure of DPH, the carbon, nitrogen, hydrogen, and oxygen are shown with cyan, blue, white, and red spheres, respectively. Also, the calculated pK value of nitrogen is determined.

Diphenhydramine hydrochloride (DPH) is the first-generation antihistamine used in medicine to treat allergic symptoms caused by the release of histamine^34,35^. Figure 1 shows the chemical structure of DPH. It has been considered as a promising corrosion inhibitor due to the presence of active centers such as the heteroatoms N and O, as well as the presence of two aromatic rings in the compound. On the other hand, its cheapness, availability, and environmentally friendly properties can make it an excellent alternative for expensive and toxic inhibitors.

In this study, expired DPH drugs were employed as a novel corrosion inhibitor for MS in a 1 M HCl medium for the first time. Previously it was claimed that a comparison of surface coverage data determined by EIS methods could be used to estimate molecules' orientation on the surface with no proof. For the first time, this paper provides clear evidence based on the molecular dynamic (MD) method for such comparison. To this end, various electrochemical techniques such as EIS and PDP were used to explore the inhibition properties of DPH. Field emission-scanning electron microscopy (FE-SEM)/energy dispersive X-ray (EDX), atomic force microscopy (AFM), and other techniques were used to analyze the metal surface. Finally, chemical quantum calculations (QM) at different concentrations were performed using density-functional theory (DFT) and MD simulation. We believe that this work will shed new light on the use of experimental studies and chemical quantum analysis to precisely evaluate the influence of concentrations of corrosion inhibitors in preventing metal corrosion.

## Experimental

### Materials and sample preparation

As the working electrode, 10 × 3 × 0.1 cm^3^ of St12 steel was procured from Mobarakeh (Iran). HCl (37%) was purchased from Mojallali (Iran) and diluted to 1 M using deionized water. DPH was purchased from Amin Pharmaceutical Company (Iran) at a concentration of 2500 ppm and subsequently diluted to 250, 500, 750, and 1000 ppm. It is noteworthy that DPH is freely soluble in water and HCl (solubility > 100 g/L). In order to prepare the MS surface for electrochemical tests, it was washed with acetone, sanded with 400–1200 grid size SiC paper, and then washed again with acetone.

### Electrochemical tests

A 1 × 1 cm^2^ area of MS was submerged in the electrolyte containing different concentrations of DPH, and the rest of the surface was insulated using colophony/beeswax mixture (1.3:3 w:w%). Electrochemical tests were performed using the CorrTest electrochemical workstation (CS350, China). MS plate, platinum, and saturated calomel (3 M KCl) were selected as working, counter, and reference electrodes. EIS was carried out in the frequency range of 10 mHz to 10 kHz with an amplitude of 10 mV at open circuit potential (OCP). PDP was also accomplished after 24 h in the potential range of − 250 to + 250 mV vs. OCP (scan rate = 0.5 mV/s). The assessments were performed three times to ensure the correctness of the results.

### Surface analyses

Surface analyses were performed by soaking an MS plate with dimensions of 1 × 1 × 0.1 cm^3^ for 6 h in acid solution without DPH and with a 1000 ppm DPH. After that, it was rinsed with deionized water and dried at 60 °C in an oven. FE-SEM (Mira (III) TE-Scan) with a 15 kV field emission source (Oxford Instruments EDX Microanalysis X-MAX-80) were utilized to examine the morphology and elements on the MS surface. AFM (BRUKER, ICON, United States) was used to study the surface topology. X-ray photoelectron spectroscopy (XPS, BESTEC EA10, Germany), grazing incidence X-ray diffraction (GIXRD, PHILIPS, PW1730, Netherlands), and Fourier-transform infrared spectroscopy (FTIR, THERMO, AVATAR, United States) analyses were also used to investigate the surface layer composition formed on MS. The adsorption of DPH on the metal surface was studied using ultraviolet–visible spectroscopy (UV–Vis, THERMO, BIOMATE5, United States). Hydrophobicity/hydrophilicity and surface chemistry of MS surface was also explored using a contact angle device.

### Computational study

#### QM calculations

QM calculations were performed to evaluate the electronic properties of DPH using Gaussian 09. Figure 1 demonstrates the chemical structure of DPH. The pK value of nitrogen atom in DPH is 8.87 calculated by MarvinSketch 18.10 software. Due to the protonation of the nitrogen atom, the DPH has one positive charge in the pH range from 1 to 7.5. By increasing pH from 7.5 to 11, the positive charge of DPH decreases. Furthermore, when pH varies from 11 to 14, DPH has zero charge. The DFT, B3LYP/6-31G(d,p) model^36^ with the electrostatic potential (ESP) method^37^ was employed for the computation of the highest occupied molecular orbital (HOMO), lowest unoccupied molecular orbital (LUMO), and partial atomic charges. The reactivity of DPH was assessed with the electronegativity ($\chi$), hardness ($\eta$), and a fraction of transferred electrons ($\Delta N$) which are defined in Eqs. (1)–(3)^38–41^. These parameters relate to the intrinsic ionization potential (*I* = − *E*_*HOMO*_) and electron affinity (*A* = − *E*_*LUMO*_).

1 $$\chi = \frac{{{\text{I}} + A}}{2} = - \frac{{E_{HOMO} + E_{LUMO} }}{2}$$

2 $$\eta = \frac{{{\text{I}} - A}}{2} = \frac{{E_{LUMO} - E_{HOMO} }}{2} = \frac{{\Delta E_{L - H} }}{2}$$

3 $$\Delta N = \frac{{\chi_{Fe} - \chi_{Inhibitor} }}{{2\left( {\eta_{Fe} + \eta_{Inhibitor} } \right)}} = \frac{{\phi - \chi_{Inhibitor} }}{{2\eta_{Inhibitor} }}$$

The work function of MS with a value of 4.5 eV was utilized instead of its electronegativity to compute the proportion of transported electrons, and the MS hardness was ignored^39,42,43^. The nucleophilic, electrophilic, and radial Fukui functions are represented in Eqs. (4), (5), and (6), respectively, where the $\rho_{N - 1} \left( {\vec{r}} \right)$, $\rho_{N} \left( {\vec{r}} \right)$, and $\rho_{N + 1} \left( {\vec{r}} \right)$ express the charge densities of anionic, neutral, and cationic molecules, respectively and $f^{ + } \left( {\vec{r}} \right)$, $f^{ - } \left( {\vec{r}} \right)$, and $f^{0} \left( {\vec{r}} \right)$ signify the nucleophilic, electrophilic, and radical attacks.

4 $$f^{ + } \left( {\vec{r}} \right) = \rho_{N + 1} \left( {\vec{r}} \right) - \rho_{N} \left( {\vec{r}} \right)$$

5 $$f^{ - } \left( {\vec{r}} \right) = \rho_{N} \left( {\vec{r}} \right) - \rho_{N - 1} \left( {\vec{r}} \right)$$

6 $$f^{0} \left( {\vec{r}} \right) = \rho_{N + 1} \left( {\vec{r}} \right) - \rho_{N - 1} \left( {\vec{r}} \right)]/2$$

#### MD simulation

##### The structural model

The Fe crystal (110), which has 432 atoms in a thickness of 15 Å, was made to represent the MS^44^. The choice of Fe (110) crystalline surface was based on the composition of MS, which is approximately 99% iron^44^. Also, The comparison between different surfaces has verified that surfaces with miller indices of (110) are thermodynamically stable and have high packed surfaces^42,45^.

##### Force field

All simulations performed with CHARMM force field^46^ and simple point charge (SPC) model was considered for water molecules^47^. The Lennard–Jones parameters for the interaction of MS atoms were taken from the reference^48^.

##### MD simulation

Simulations were accomplished using the GROMACS 5.1.4 MD software. The temperature was maintained at 298 K using a V-rescale thermostat^49^ with a coupling time constant of 0.5 ps. The Berendsen method^50^ was used to keep the simulation box pressure at 1 bar. The equation of motion with a time step of 1 fs was integrated with the leap-frog algorithm^51^ under periodic boundary conditions in x,y, and z directions.

As reported in Table 1, four simulation boxes were evaluated to track the molecular interactions between DPH and MS. DPH molecules were distributed haphazardly around the MS crystal at each concentration, and the simulation box was filled with water molecules and HCl with a 1 M concentration. The MS crystal in acid solution without DPH was considered as a referenced system.

**Table 1:** The components of the simulation systems.

DPH concentration (ppm)	Number of DPH molecules	Number of water molecules	Box dimensions (nm^3^)
0	0	31,762	9.94 × 9.94 × 9.94
250	1	54,747	11.90 × 11.90 × 11.90
500	1	31,659	9.94 × 9.94 × 9.94
750	2	31,644	9.94 × 9.94 × 9.94
1000	3	31,630	9.94 × 9.94 × 9.94

The linear constraint solver algorithm (LINCS) was applied to constrain all bonds^52^. The cutoff radius of Lennard–Jones (LJ) and electrostatic interactions were set to 1.2 nm. The particle-mesh Ewald (PME) summation^53^ was used to compute electrostatic interactions, and the neighbor list was updated every 10 fs via searching. After guaranteeing that the simulation has reached equilibration, by energy minimization, the temperature was maintained constant in the NVT ensemble for 10 ns, and then pressure and density were kept constant in the NPT ensemble for 10 ns. The MD run period for each simulation was 30 ns, and the last 10 ns of the MD step was used for study. Molecular visualization was executed with the visual molecular dynamics (VMD 1.9.1) program.

## Results and discussion

### Inhibition action

**Figure 2:**
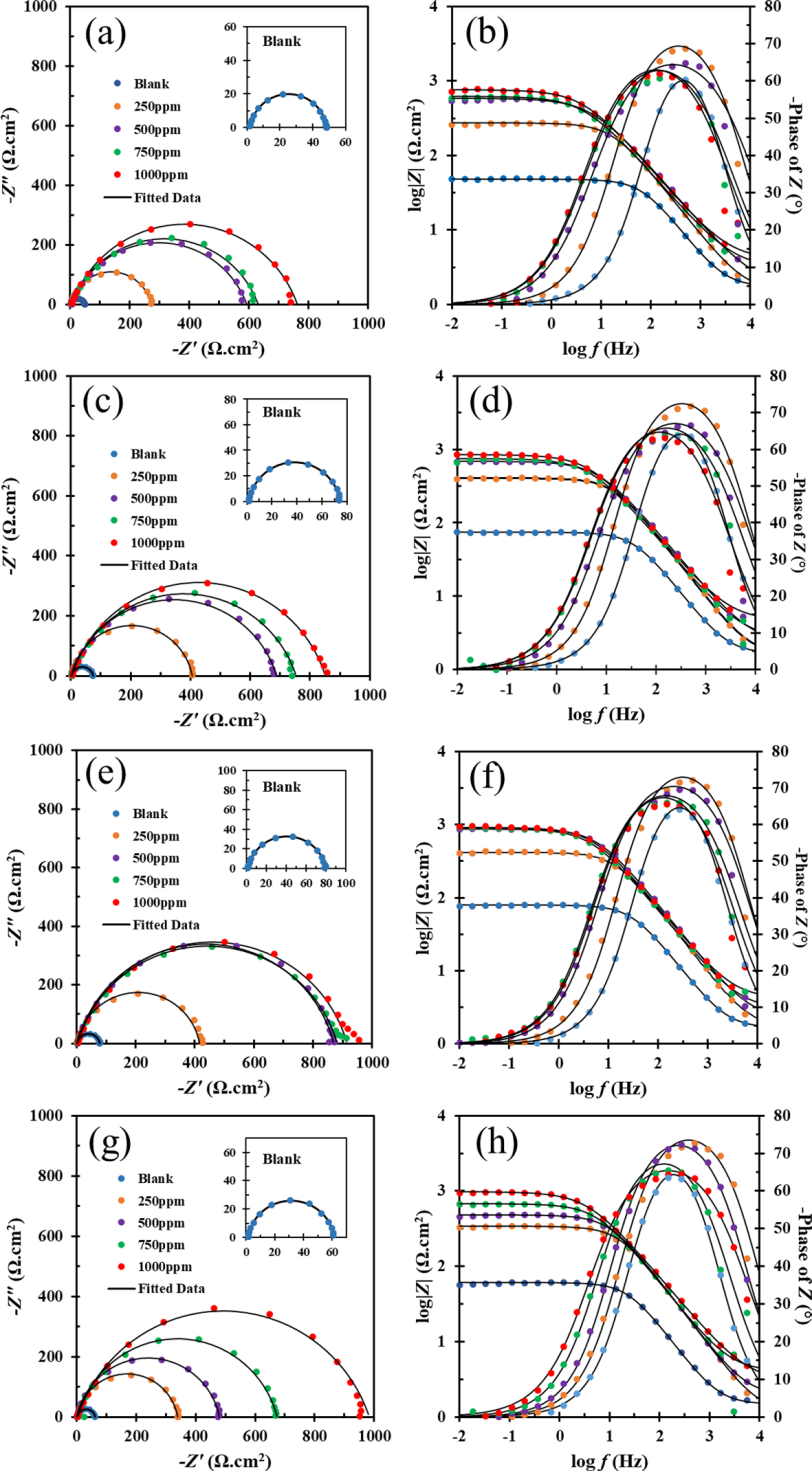
Nyquist (left) and Bode (right) diagrams for prepared samples after 1 h (**a**,**b**), 3 h (**c**,**d**), 6 h (**e**,**f**) and 24 h (**g**,**h**) immersion in 1 M HCl solution containing various concentration of DPH at room temperature.

Evaluation of the corrosion prevention capabilities of DPH was carried out using the EIS method. Figure 2 depicts the Nyquist, and Bode graphs of MS dipped in a solution without and with various concentrations of DPH. The diagrams depict the behavior of one time-constant circuit. As a result, charge transfer controls the corrosion reaction^54^. The figures clearly show that the blank sample has a semicircle with a significantly smaller diameter than that of the inhibitor-treated samples. These observations indicate severe corrosion of MS in the acidic environment resulting from the dissolution of MS or the destruction of the oxide film formed on the metal surface.

Different amounts of DPH inhibitor were added to the solution in order to evaluate the corrosion behavior of MS. Thanks to the addition of DPH, as seen in Fig. 2, the diameter of the semicircle of the Nyquist diagram rises, which shows the adsorption of inhibitor active chemicals onto the metal surface. On the other hand, the increment of the DPH concentration enhanced the charge transfer resistance, which is related to the development of more dense and compact films on the steel surface. The value of impedance at the lowest frequency (|*Z*|_0.01 Hz_) indicates that the overall corrosion resistance has increased with increasing DPH concentrations. Additionally, when the DPH concentration increases, the maximum phase angle moves to lower frequencies, demonstrating the development of a shielding layer on the MS surface^55^. For more detailed analysis and obtaining impedance parameters, the EIS data was fitted with an electrical circuit with one time constant (R(Q(R)) (Supplementary Information, Fig. S1). Table 2 shows the parameters of the fitted electrical circuit. In this table, *R*_*s*_, *R*_*ct*_, *CPE*, *C*_*dl*,_ and *θ*_*EIS*_ signify the solution resistance, charge transfer resistance, constant phase element, double-layer capacitance, and surface coverage (based on EIS analysis), respectively. Also, *Y*_*0*_ represents admittance, *n* reflects the power of the constant phase element, and *τ* is the time constant. Due to the non-ideal surface of the capacitor and the presence of surface roughness, a constant phase element has been used. The *C*_*dl*_, *η*, *τ*, and *θ*_*EIS*_ were calculated using Eqs. (7), (8), (9), and (10), respectively. *R*_*ct,0*_ and *R*_*ct,i*_ represent charge transfer resistance in the absence and presence of DPH, respectively. In addition, *C*_*dl,i*_ denotes double-layer capacitance in the presence of DPH, whereas *C*_*dl,0*_ signifies double-layer capacitance without DPH.

7 $$C_{dl} = Y_{0}^{\frac{1}{n}} \times \left( {\frac{{R_{s} \times R_{ct} }}{{R_{s} + R_{ct} }}} \right)^{{\frac{1 - n}{n}}}$$

8 $$\eta \;\% = \left( {1 - \frac{{R_{ct. 0} }}{{R_{ct. i} }}} \right)100\%$$

9 $$\tau = R_{ct} \times C_{dl}$$

10 $$\theta_{EIS} = \left( {1 - \frac{{C_{dl.i} }}{{C_{dl.0} }}} \right)100\%$$

**Table 2:** EIS parameters of MS in the presence of different concentrations of DPH in 1 M HCl solution.

Concentration (ppm)	Immersion time (h)	*R*_*s*_ (Ω cm^2^)	*R*_*ct*_ (Ω cm^2^)	*CPE*		*C*_*dl*_ (µF cm^−2^)	*η* (%)	*τ* (s)	*θ*_*EIS*_
				*Y*_*0*_ (µs^n^ Ω^−1^ cm^−2^)	*n*				
Blank	1	1.71 ± 0.01	46.75 ± 0.17	92.25 ± 1.82	0.9 ± 0.01	34.73	–	0.00162	–
	3	1.65 ± 0.01	72.84 ± 0.24	104.86 ± 1.34	0.89 ± 0.01	35.85	–	0.00261	–
	6	1.65 ± 0.01	77.88 ± 0.3	117.59 ± 1.78	0.89 ± 0.01	40.78	–	0.00317	–
	24	1.49 ± 0.01	60.04 ± 0.42	175.2 ± 4.12	0.9 ± 0.01	69.86	–	0.00419	–
250	1	1.51 ± 0.07	271.1 ± 4.21	63.6 ± 2.96	0.86 ± 0.01	14.09	82.75	0.00382	59
	3	1.59 ± 0.09	404.4 ± 6.32	46.3 ± 2.16	0.88 ± 0.01	12.64	81.99	0.00511	64
	6	1.72 ± 0.11	413.8 ± 9.25	44.5 ± 2.72	0.89 ± 0.01	13.78	81.17	0.00570	66
	24	1.19 ± 0.09	338.3 ± 5.98	49.2 ± 2.67	0.89 ± 0.01	14.78	82.25	0.005	78
500	1	1.74 ± 0.2	592.6 ± 12.04	92.86 ± 4.44	0.78 ± 0.01	7.91	92.11	0.00468	77
	3	2.3 ± 0.18	685.1 ± 9.19	65.96 ± 2.54	0.81 ± 0.01	8.38	89.37	0.0057	76
	6	2.25 ± 0.19	867.2 ± 12.77	53.55 ± 2.27	0.84 ± 0.01	9.55	91.02	0.0082	76
	24	1.93 ± 0.05	478.2 ± 4.26	53.74 ± 1.26	0.88 ± 0.01	15.37	87.44	0.00735	77
750	1	3.08 ± 0.22	628 ± 12.72	111.19 ± 7.70	0.78 ± 0.01	11.70	92.55	0.00734	66
	3	2.68 ± 0.19	751.3 ± 13.75	85.97 ± 3.60	0.8 ± 0.01	10.58	90.30	0.0079	70
	6	2.4 ± 0.20	877.5 ± 14.68	73.5 ± 3.21	0.82 ± 0.01	11.02	91.12	0.00966	72
	24	4.03 ± 0.24	670.8 ± 9.6	73.19 ± 3.18	0.84 ± 0.01	15.54	91.05	0.01042	70
1000	1	4.52 ± 0.26	748.2 ± 16.36	89.78 ± 4.55	0.8 ± 0.01	12.72	93.75	0.0095	63
	3	4.66 ± 0.24	846.9 ± 0.24	77.37 ± 3.3	0.81 ± 0.01	12.03	91.40	0.01019	66
	6	4.18 ± 0.24	909.1 ± 13.35	64.95 ± 3.30	0.83 ± 0.01	12.07	91.43	0.01097	70
	24	2.99 ± 0.09	977.5 ± 9.70	82.82 ± 1.94	0.79 ± 0.01	9.10	93.86	0.00889	86

According to the Nyquist diagram and Table 2, it can be seen that at the beginning of immersing the MS in the blank solution (1 h), the diameter of the semicircle was small, and then with the passage of time up to 6 h, the diameter of the semicircle and the *R*_*ct*_ increased. The buildup of corrosion products with semi-protective characteristics may be responsible for the increase in diameter^56^. According to Table 2, after 6 h of immersion, the *R*_*ct*_ increased with increasing DPH concentration and reached 909.1 Ω cm^2^ at a concentration of 1000 ppm. Also, by comparing the percentage of inhibition efficiency, it can be seen that at concentrations above 500 ppm, the *η* has changed slightly to about 91%, which indicates a high level of efficiency in 6 h. After 24 h, the *R*_*ct*_ of MS submerged in 250, 500, and 750 ppm DPH solutions decreases, which may be attributed to corrosive species penetrating and destroying the protective layer.

On the other hand, the *R*_*ct*_ of the 1000 ppm DPH solution has increased. This might be owing to the existence of more protective species on the metal, as well as the increased compactness of the protective layer that has been adsorbed onto the metal surface^10^. The parameter “*n*” may be used to analyze surface roughness, where *n* = 0 indicates resistance, *n* = + 1 suggests ideal capacitor, and *n* = − 1 implies inductor. Comparison of parameter “*n*” in different concentrations of DPH shows slight changes compared to the blank solution, which can be owing to the adsorption of DPH on the MS surface and, consequently, change in surface heterogeneity. The reduction in *Y*_*0*_ and *C*_*dl*_ for MS immersed in DPH solutions compared to the blank sample is affected by the substitution of water molecules and DHP inhibitors, which, in addition to lowering the dielectric coefficient, increases the distance between the capacitor plates. Also, as expected, the time constant for samples with inhibitor is higher than those immersed in the blank solution, and the time constant has grown with increasing inhibitor concentration, indicating the inhibitor's immediate adsorption process^57^.

EIS analysis can offer highly fascinating information regarding the orientation of the inhibitor molecule on the surface of MS, in addition to providing helpful information about corrosion inhibition action. According to the literature, if *η*% > *θ*_*EIS*_, the inhibitor molecule is adsorbed horizontally and if *η*% < *θ*_*EIS*_, the inhibitor molecule is adsorbed vertically on the surface^58–60^. This can be explained by the fact that the inhibitor's horizontal adsorption might increase surface blockage, resulting in an increment in charge transfer resistance and *η*%. The vertical adsorption of the inhibitor, on the other hand, increases the thickness of the double layer while only blocking a small portion of the surface. As a result, it has a negligible impact on charge transfer resistance. Table 2 shows that inhibition efficiency is higher than surface coverage at all concentrations. As an outcome, the DPH molecules horizontally adsorb on the MS surface. These findings are consistent with MD simulations. Supplementary Information contains videos of DPH adsorption on MS surface (Videos S1, S2, S3, and S4).

**Figure 3:**
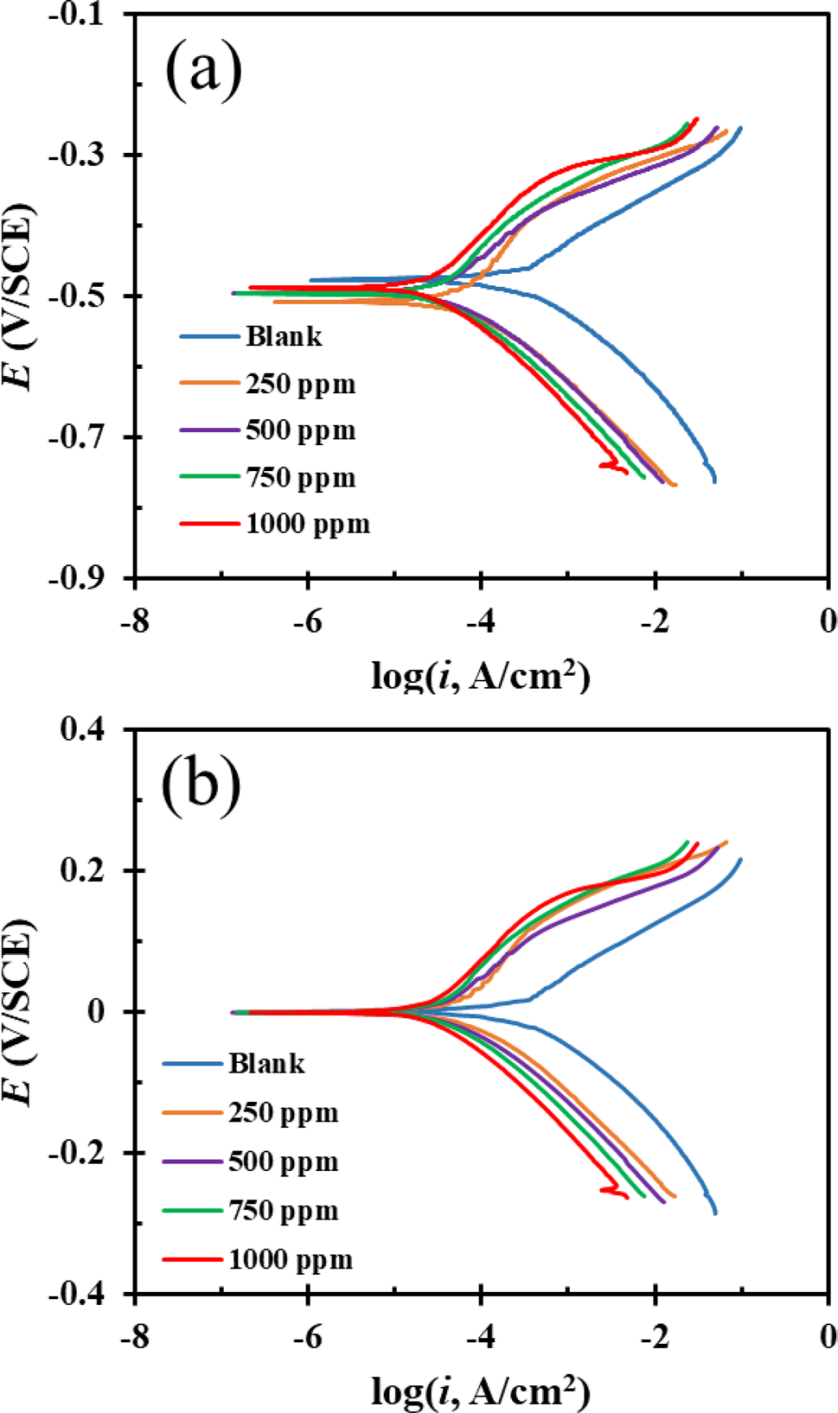
PDP curves after 24 h immersion of MS in 1 M HCl solution without DPH and with different concentrations of DPH at room temperature (**a**) PDP curves shifted to E = 0 (**b**).

The PDP test was used to explore the kinetics of anodic and cathodic reactions as well as the mechanism of DPH inhibition. Figure 3a depicts the PDP diagram obtained after immersing the MS plate in a solution without inhibitor and a solution with inhibitor at various concentrations for 24 h. In addition, for a more accurate comparison, all curves are adjusted to *E* = 0 and presented in Fig. 3b.

Table 3 shows the electrochemical polarization parameters obtained by extrapolating the Tafel slopes in the anodic and cathodic branches, where *E*_*corr*_ denotes the corrosion potential, *i*_*corr*_ symbolizes the corrosion current density, and *βc* and *βa* denotes the anodic and cathodic branch slopes, respectively. In addition, the percentage of inhibition efficiency based on PDP test (IE %) was calculated using Eq. (11). *i*_*corr,i*_ and *i*_*corr,0*_ represent corrosion current density without and with DPH, respectively.

11 $$IE \% = \left( {1 - \frac{{i_{corr.i} }}{{i_{corr.0} }}} \right) 100\%$$

**Table 3:** PDP parameter obtained from extrapolation of Tafel curves for MS plates immersed in 1 M HCl solution with various concentrations of DPH after 24 h.

	*βa* (V)	*βc* (V)	*i*_*0*_ (µA/cm^2^)	*E*_*0*_ (V)	*IE* (%)	*θ*_*PDP*_
Blank	0.0688	− 0.0711	194.0885	− 0.47589	–	–
250 ppm	0.1647	− 0.0868	60.255	− 0.50679	68.954	0.689
500 ppm	0.1109	− 0.0662	30.5492	− 0.49355	84.260	0.842
750 ppm	0.1175	− 0.07813	28.1190	− 0.49537	85.512	0.855
1000 ppm	0.1327	− 0.0966	26.0015	− 0.48716	86.603	0.866

According to the PDP curve, it is noticed that *i*_*corr*_ considerably decreased with increasing DPH concentration as compared to the blank form, and the shape of the curve also altered. Upon closer inspection of the cathodic branch, it can be seen that the diagrams in this section have a similar form and are nearly parallel to one another, indicating that the cathodic reaction mechanism has not changed as a result of the addition of DPH (2H^+^  + 2e^−^ → H_2_). A small change in the slope of the cathode branch suggests that the cathode reaction is controlled by activation^61^. On the other hand, the addition of DPH causes variations in the anodic branch, indicating an alteration in the anodic reaction mechanism, which can be explained by the presence of hydrated chloride ions or the adsorption of DPH molecules^62^. The polarization test indicates that the sample submerged in a solution containing 1000 ppm of DPH exhibits the highest level of inhibition. In this case, the current density is reduced from 194 µA/cm^2^ (blank solution) to 26 µA/cm^2^ for the 1000 ppm DPH solution, suggesting the adsorption of active substances in the DPH molecule on the MS surface and blocking the activated spots. These observations are in agreement with the findings of the EIS analysis. *E*_*corr*_ comparisons of inhibitor-treated and blank samples reveal a maximum difference of 31 mV. According to literature, if the difference in *E*_*corr*_ of MS in the existence and lack of an inhibitor is larger than 85 mV, the inhibitor acts as anodic or cathodic inhibitor, and if it is less than 85 mV, the inhibitor acts as a mixed type^10,63,64^. Therefore, DPH has a mixed inhibition mechanism for MS in 1 M HCl solution.

As an outcome, DPH can be considered as a potent corrosion inhibitor acting through adsorption on the MS surface, producing a film on the metal surface.

### Adsorption mechanism

As mentioned in the previous sections, the inhibitor plays a vital role in protecting metals against corrosion by adsorption on the MS surface and creating physical or chemical interactions with the metal surface. More in-depth research on this issue is being conducted using adsorption isotherm models. In other words, one of the approaches to model the adsorption/desorption mechanism of the inhibitor on the MS surface is to utilize adsorption isotherms. In this study, various models including Temkin, Freundlich, Frumkin, and Langmuir have been used to investigate the adsorption behavior of DPH, which are expressed by the following equations (Eqs. 12–15):

12 $${\text{Temkin:}}\quad \exp \left( { - 2\alpha \theta_{PDP} } \right) = K_{ads} C_{DPH}$$

13 $${\text{Freundlich:}}\quad \theta_{PDP} = K_{ads} C_{DPH}^{n }$$

14 $${\text{Frumkin:}}\quad \frac{{\theta_{PDP} }}{{1 - \theta_{PDP} }}\exp \left( { - 2\alpha \theta_{PDP} } \right) = K_{ads} C_{DPH}$$

15 $${\text{Langmuir:}}\quad \frac{{C_{DPH} }}{{\theta_{PDP} }} = \frac{1}{{K_{ads} }} + C_{DPH}$$

**Figure 4:**
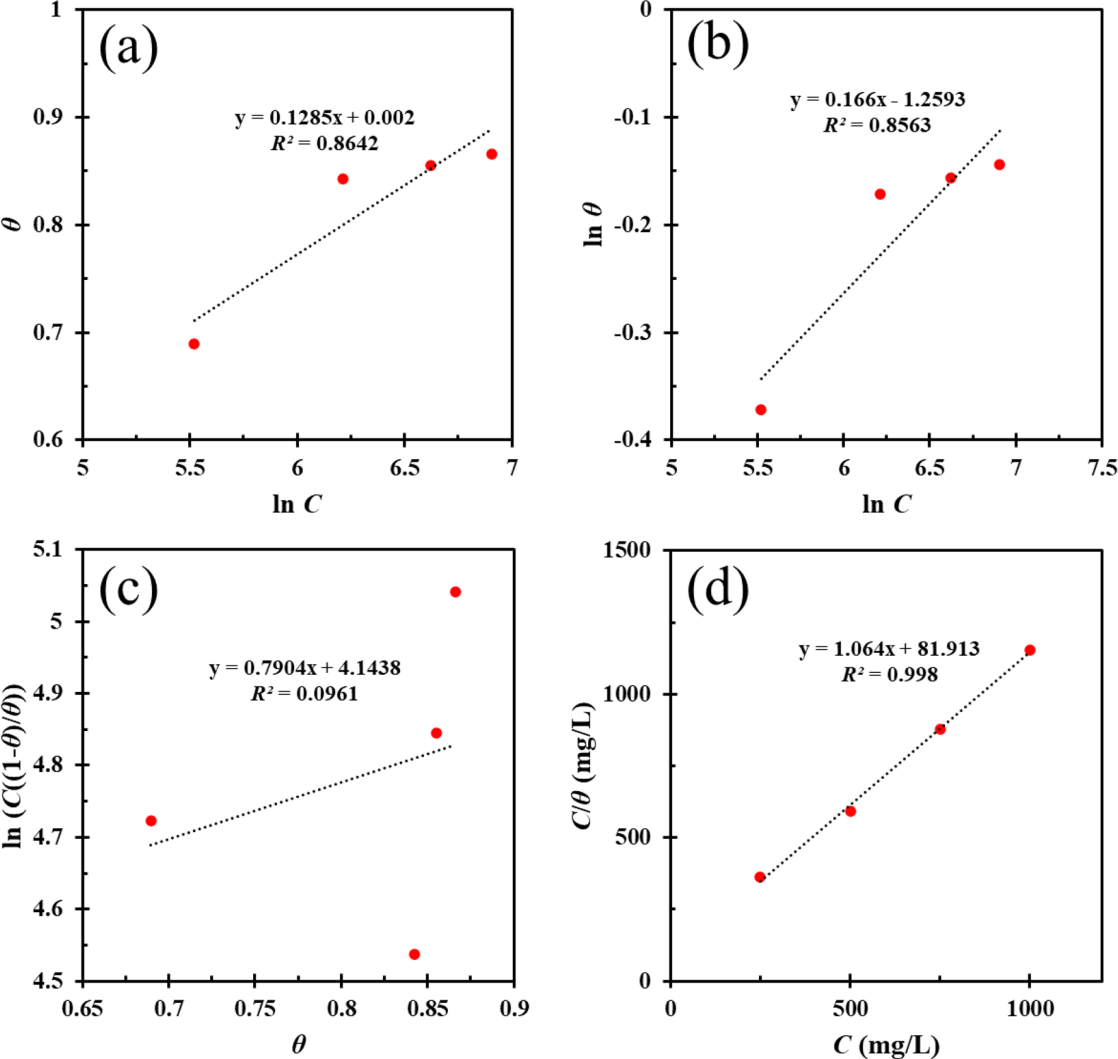
Temkin (**a**), Freundlich (**b**), Frumkin (**c**), and Langmuir (**d**) isotherms used for studying adsorption of DPH on MS surface after 24 h immersion of MS in 1 M HCl solution containing 0–1000 ppm DPH at room temperature.

In the above equations, *C*_*DPH*_ is DPH concentration, *K*_*ads*_ represents the equilibrium adsorption/desorption constant, *α* denotes the lateral interaction of inhibitor-surface and $\theta_{PDP} = \frac{IE\% }{{100}}$ stands for surface coverage based on PDP analysis. As shown in Fig. 4, the Langmuir model provides the best fit for the experimental data, and the *R*^2^ value (coefficient of correlation value) validates this. This model suggests that DPH molecules have no interaction with each other, and also their adsorption on the MS surface is monolayer^65–67^. *K*_*ads*_ can be obtained by drawing *C*_*DPH*_/$\theta_{PDP}$ versus *C*_*DPH*_. Additionally, the Gibbs free energy change $\Delta G_{ads}^{0}$ is correlated to *K*_*ads*_ via Eq. (16), where R is the universal gas constant, T is the temperature in Kelvin, and 55.5 is the molar value of water in solution. The values for K_ads_ and $\Delta G_{ads}^{0}$ are given in Table 4.

16 $$\Delta G_{ads}^{0} = - RT \times \ln \;\left( {55.5K_{ads} } \right)$$

**Table 4:** Langmuir isotherm parameters for DPH adsorbed onto MS surface submerged in 1 M HCl solution containing 0–1000 ppm DPH at 298 K.

$\Delta G_{ads}^{0}$(kJ/mol)	*K*_*L*_ (l/mg)	*R*^2^
− 21.35	0.0122	0.998

According to Table 4, the negative value for $\Delta G_{ads}^{0}$ suggests that the DPH adsorption progression on the MS surface is spontaneous. If the value of $\Delta G_{ads}^{0}$ is less than − 40 kJ/mol, it shows the chemical adsorption, while the values exceeding − 20 kJ/mol suggests the physical adsorption^64^. In this case, the quantity of free energy of adsorption is − 21.35 kJ/mol, indicating that physical and chemical adsorption are occurring concurrently.

Table 5 compares the corrosion inhibition of several medicines employed as corrosion inhibitors in HCl media. This table highlights the characteristics of corrosion inhibition efficiency at the optimum concentration (based on EIS study) as well as the adsorption isotherm. As demonstrated, DPH is one of the most effective corrosion inhibitors, with a corrosion efficiency of greater than 90%. Also, its low cost and wide availability make it an attractive candidate for mitigating MS corrosion.

**Table 5:** Comparison of corrosion inhibition efficiency and adsorption isotherm of different drugs in 1 M HCl medium.

Drug	Optimum concentration (ppm)	Inhibition efficiency at the optimum concentration (%)	Adsorption isotherm	Ref
Cefalozin	500*	93.9	Langmuir	^20^
Pheniramine	200*	86.4	Langmuir	^23^
Streptomycin	500	83.9	Langmuir	^24^
Analgin	4000	92.47	Langmuir	^21^
Metformin hydrochloride	500	83.97	Langmuir	^25^
Penicilin G	3350*	95.9	Langmuir	^26^
Ampicilin	3500*	95.5	Langmuir	^26^
Amoxicilin	3650*	93.7	Langmuir	^26^
Cefalexin	400	90	Langmuir	^27^
Losartan potassium	2300*	88.9	Langmuir	^19^
Ketosulfone	200	77.7	Langmuir	^28^
Cefixime	400*	91.8	Langmuir	^29^
Atorvastatin	150	96.38	Langmuir	^30^
Pantoprazole sodium	300	92.2	Langmuir	^32^
Ondansetron hydrochloride	300	88.56	Langmuir	^33^
Betahistine dihydrochloride	1500	96.21	Langmuir	^68^
Isoxsuprine hydrochloride	2000	97.83	Langmuir	^69^
Diphenhydramine hydrochloride (DPH)	1000	93.86	Langmuir	Present study

*The concentration of these compounds is converted from mM to ppm.

### Inhibition mechanism

According to the preceding, the mechanism of adsorption of DPH molecules on the surface of MS is physicochemical. It is well understood that MS is positively charged in the HCl medium, so chloride ions (negatively charged) can be adsorbed on its surface^70^. The presence of chloride ions on the surface of MS can cause the physical adsorption of protonated DPH molecules^71^. On the other hand, it can be demonstrated that the existence of non-bonded electron pairs on N and O, as well as the π electrons in the aromatic ring, are responsible for the chemical adsorption of DPH on the MS surface by producing a complex with an empty Fe orbital^72^.

### FE-SEM-EDX and AFM

**Figure 5:**
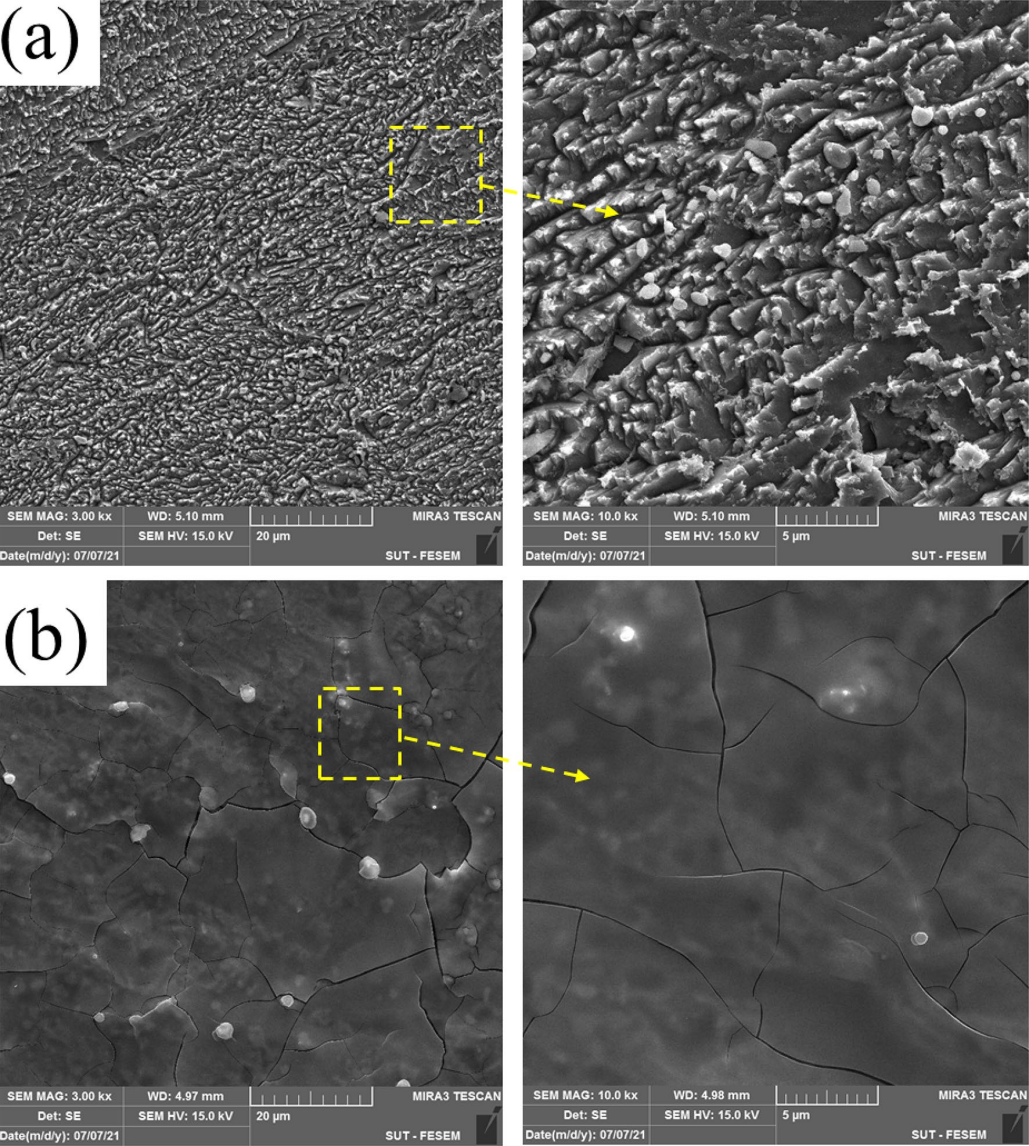
FE-SEM image of MS immersed in 1 M HCl solution without (**a**) and with 1000 ppm DPH (**b**) after 4 h.

**Figure 6:**
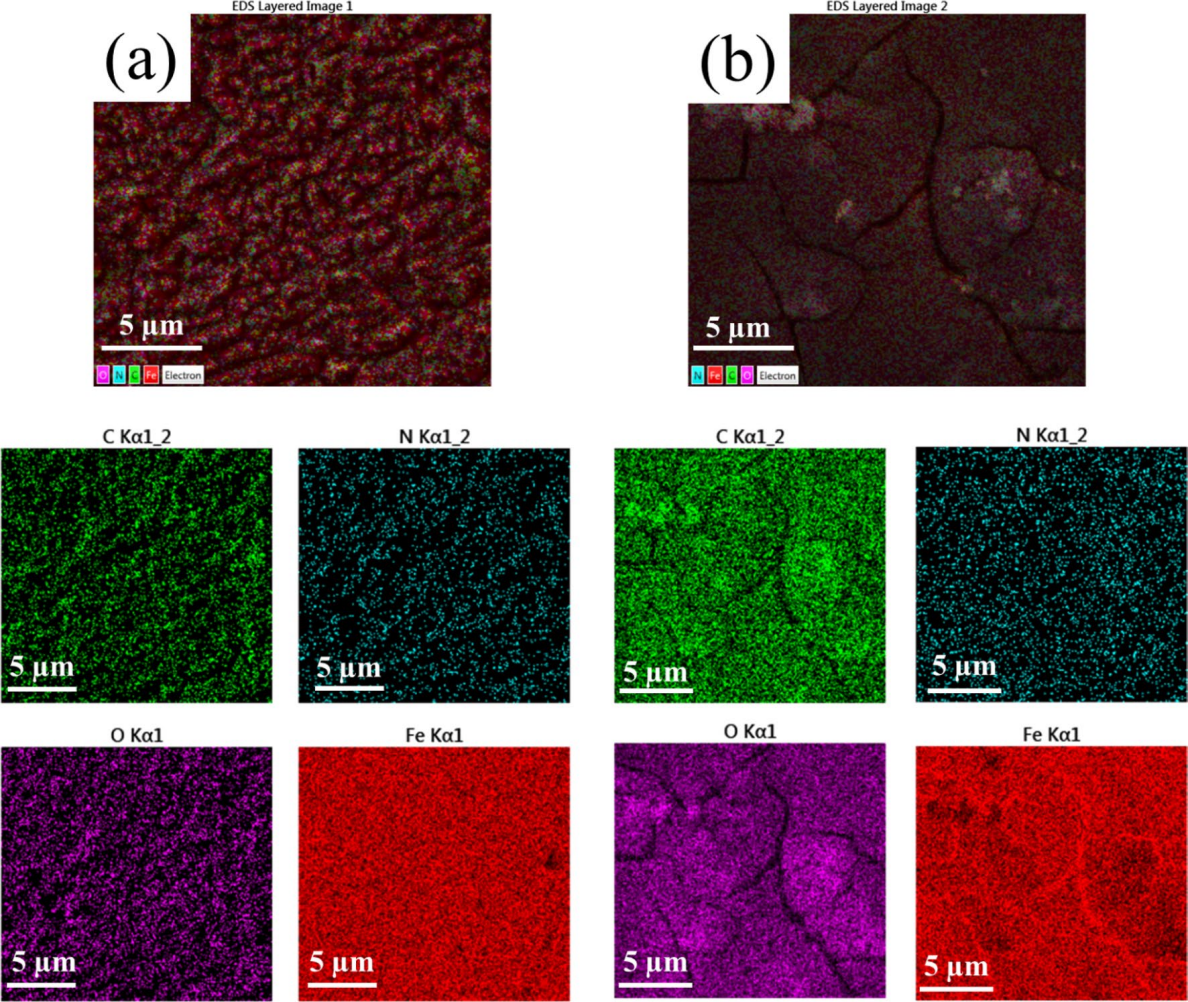
Map analysis of the MS surface immersed in 1 M HCl solution without (**a**) and with (**b**) 1000 ppm DPH after 4 h.

FE-SEM analysis was performed to analyze the surface morphology of MS submerged in 1 M HCl solution without and with 1000 ppm DPH (Fig. 5). Based on the FE-SEM image, it can be determined that the rough corrosion products have been formed on the surface of the sample exposed to the HCl solution without DPH. The formation of rough surface can be justified by the intense Cl^−^ attacks. On the other hand, the MS immersed in acid with 1000 ppm DPH shows that a protective layer is formed on the surface. This insoluble film can function as a hindrance to the passage of the corrosive species, thereby slowing down the pace of corrosion. The fractures seen in the images can be caused due to the heating operation in the oven. To validate the creation of a DPH layer on the metal surface, EDX elemental analysis was performed. The weight percentages of elements and elemental mapping of the metal surface dipped in the blank solution and a solution containing 1000 ppm of DPH are shown in Table 6 and Fig. 6, respectively. As reported in Table 6, the increase in the percentage of carbon atoms and the appearance of nitrogen atoms as well reduction in oxygen percentage indicate the production of a DPH protective layer on the metal surface, which is consistent with the prior findings. In addition, according to the literature, increasing the Fe/O ratio indicates a decrease in MS corrosion^73^. Based on EDX data, the Fe/O ratio for steel submerged in 1000 ppm DPH solution is 25.88, while for steel immersed in blank solution is 15.53. These outcomes provide more evidence of DPH's successful functioning in lowering the rate of corrosion.

**Table 6:** The weight percentage of identified elements on the surface of MS immersed in 1 M HCl solution without and with 1000 ppm DPH after 4 h.

Element	Weight percentage (%)	
	Blank	1000 ppm DPH
C	6.30	8.98
N	0.1	2.32
O	5.34	3.30
Fe	88.26	85.4

**Figure 7:**
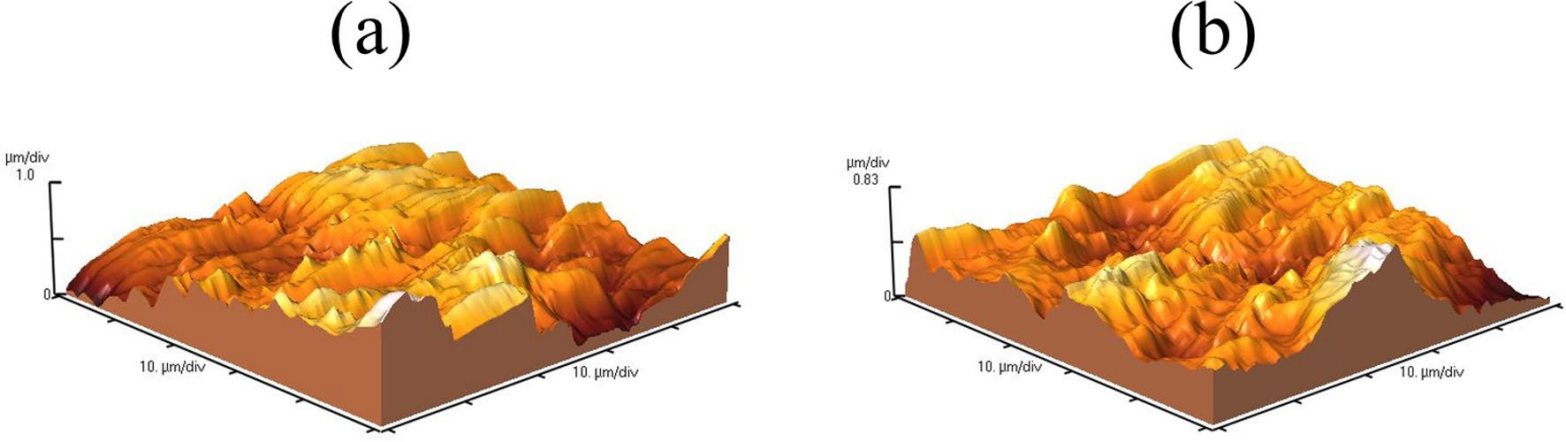
AFM analysis of MS immersed in 1 M HCl solution without (**a**) and with 1000 ppm DPH (**b**) after 4 h.

The topology of the working electrode surface was investigated using AFM analysis (Fig. 7). As shown in Table 7, the AFM analysis yielded several useful parameters, such as peak-to-valley (*R*_*p–v*_), average height (*H*_*m*_), average roughness (*R*_*q*_), and root mean square deviation (*R*_*q*_). Comparing these parameters of the sample immersed in a solution containing 1000 ppm DPH with the sample without this inhibitor, it is observed that the average roughness for the sample with inhibitor is 182.4 nm and for the blank sample is 238.8 nm. The lower roughness parameters in the presence of DPH confirm the production of a protective layer, as well as less corrosion attack on the surface.

**Table 7:** Parameters obtained from the surface of MS immersed in 1 M HCl solution without and with 1000 ppm DPH after 4 h using AFM analysis.

Sample	*R*_*p–v*_ (µm)	*R*_*a*_ (nm)	*H*_*m*_ (nm)	*R*_*q*_ (nm)
Blank	2.043	238.8	891.3	290.8
DPH (1000 ppm)	1.655	182.4	705.5	240.6

### FTIR and UV–Vis measurements

**Figure 8:**
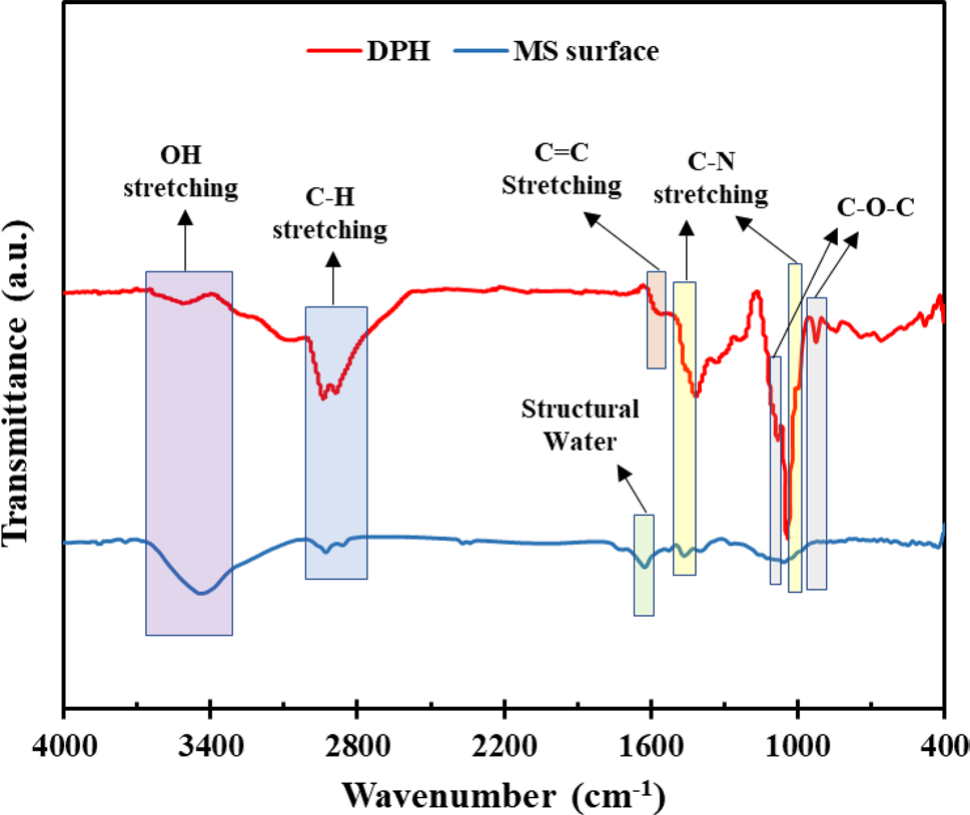
FTIR spectra of MS surface immersed in 1 M HCl solution with 1000 ppm DPH after 4 h.

The structure generated on the MS surface submerged in the inhibitor solution was studied using FTIR analysis. For comparison, FTIR analysis of the DPH solution is also presented. As seen in Fig. 8, the characteristic peaks at 3450 cm^−1^ and 1625 cm^−1^ are attributed to OH stretching and structural water^74^, respectively. The C–H stretching in the DPH molecule is responsible for the two peaks at^75,76^ 2940 cm^−1^ and 2890 cm^−1^. The C=C stretching peak appears at wavelength^77^ 1580 cm^−1^ due to the benzene rings in the DPH structure. Also, a strong peak at 1040 cm^−1^ and a weaker peak at 1460 cm^−1^ are related to C–N stretching^78^. Two peaks correlated to C–O–C stretching vibration have also appeared in wavenumbers^79,80^ 1090 cm^−1^ and 920 cm^−1^. The FTIR spectrum from the MS surface soaked in 1 M HCl solution containing 1000 ppm DPH reveals the same bonds as the DPH spectrum with a slight shift. These observations support the assumption that a protective film is created on the MS surface.

**Figure 9:**
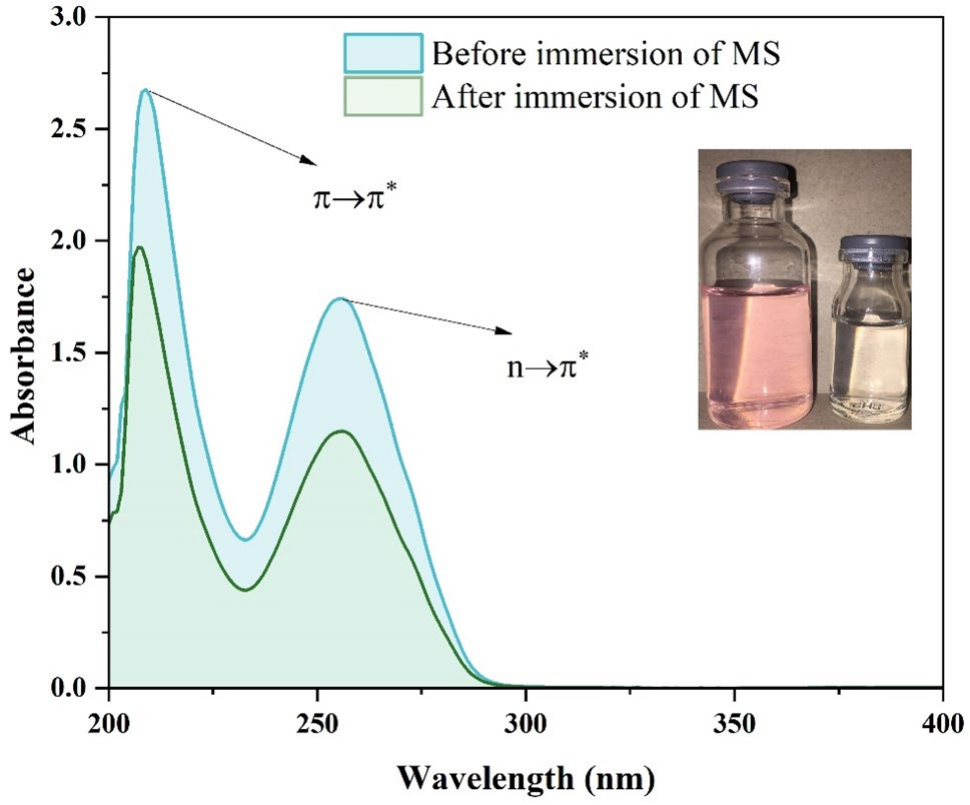
UV–Vis spectrum of 1 M HCl solution containing 1000 ppm DPH before and after 12 h immersion of MS.

UV–Vis analysis was performed to investigate the possibility of complex formation between DPH molecules and metal ions. UV–Vis spectra were recorded from 1 M HCl solution containing DPH before and after immersion of MS (Fig. 9). As shown in Fig. 9, two absorption peaks at 209 nm and 256 nm are found prior to the immersion of the MS. The absorption peak at 209 nm corresponds to π → π^*^ transition of the C=C functional groups in the DPH aromatic ring^81,82^. In contrast, the absorption peak at 256 nm can be attributed to the n → σ^*^ electronic transition of the C–O or C–N functional groups. The absorption intensity of the characteristic peaks is dramatically decreased after 12 h of immersion of MS in 1 M HCl solution containing DPH. Furthermore, the absorption peaks of 209 nm and 256 nm shifted to 207 nm and 255 nm, respectively (blue shift). The 256 nm peak shift might be due to n-cation interaction caused by nitrogen and oxygen heteroatoms, which can interact with the vacant orbital of iron ions via electron sharing. On the other hand, the interaction of the π-cation of the aromatic ring of the DPH molecule with the iron ion might explain the shift at 209 nm. The results described above provide conclusive proof of the feasibility of the creation of metal-inhibitor complexes.

### GIXRD

**Figure 10:**
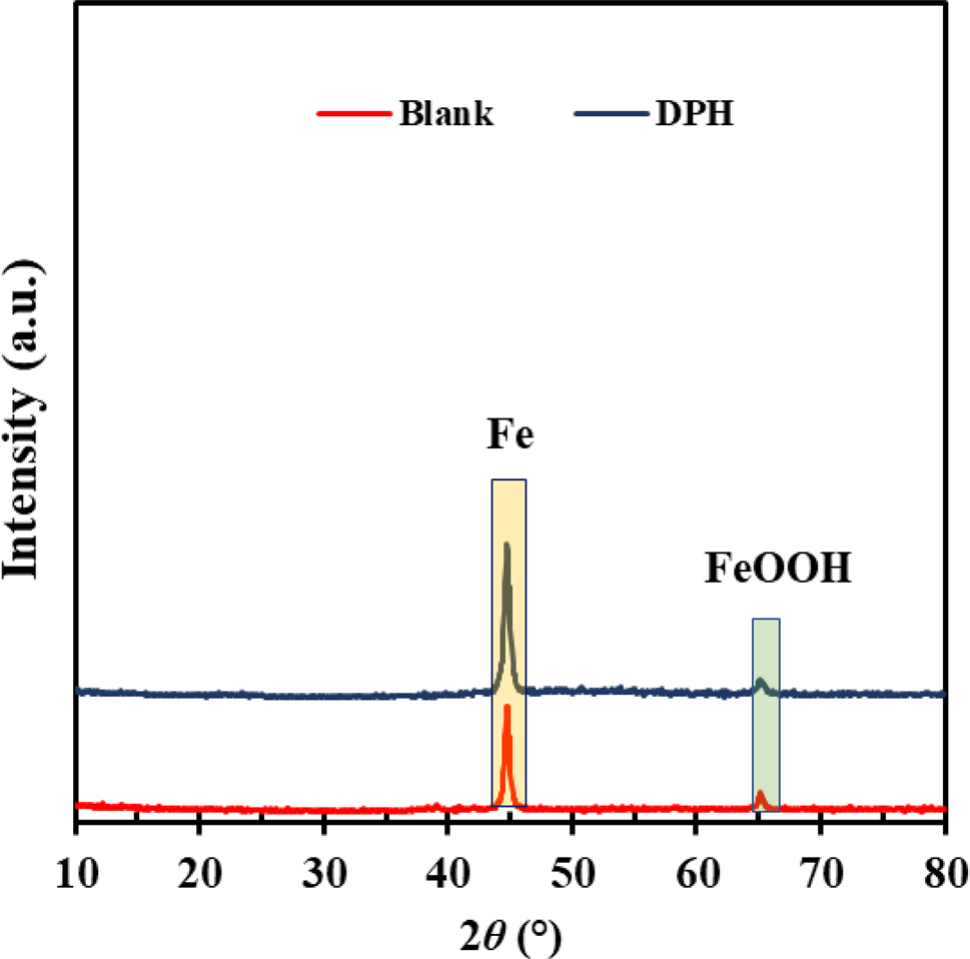
GIXRD spectra of the MS surface submerged in 1 M HCl solution without and with 1000 ppm DPH after 4 h.

GIXRD spectrum of the MS surface soaked in 1 M acid without DPH and with 1000 ppm DPH is shown in Fig. 10. Two peaks are observed in the blank sample at 2*θ* = 44.87° and 2*θ* = 64.92° corresponding to Fe metal and Lepidocrocite (γ-FeOOH)^11^. The metal surface analysis reveals an increase in the peak intensity of Fe and a reduction in the peak intensity of γ-FeOOH after adding DPH to the acidic solution. We assumed that upon the formation of an inhibitive layer on the MS surface, the corrosion reaction has been decreased, leading to the formation of fewer corrosion products and therefore more X-ray diffractions from the base metal.

### XPS

**Figure 11:**
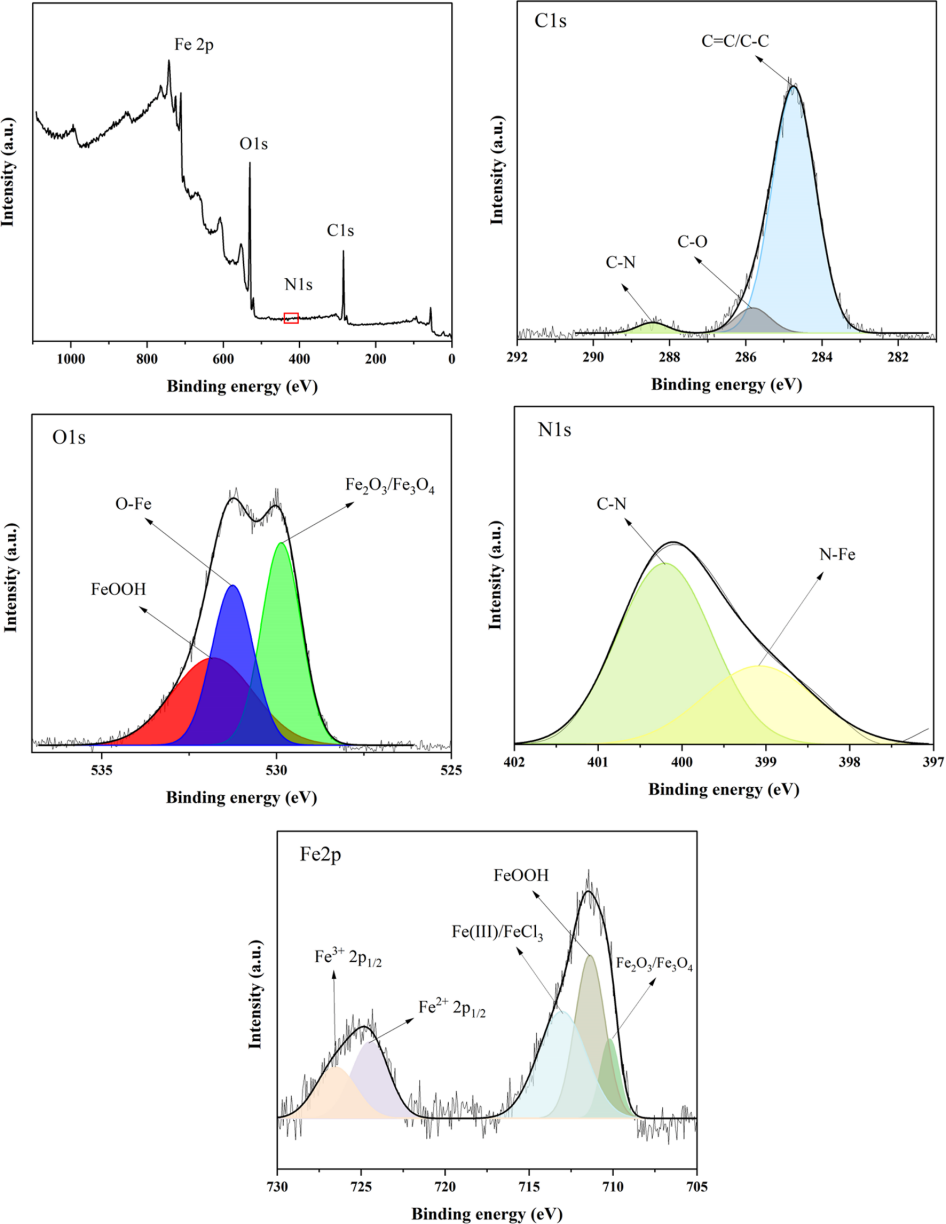
XPS survey and high-resolution spectra from the surface of MS immersed in the 1 M HCl solution containing 1000 ppm DPH after 4 h.

XPS analysis is a robust technique for investigating the bonds generated on the metal surface. Figure 11 depicts the overall XPS survey and high-resolution spectra of the major elements present on the steel surface after 6 h of soaking in the 1 M HCl solution containing 1000 ppm DPH.

The C1s spectrum consists of three main peaks. The peak observed at 284.7 eV indicates the C=C/C–C bond^83^ due to the aromatic ring in the DPH structure. Furthermore, the peaks with binding energies 285.8 eV and 288.34 eV are associated with C–O^19^ and C–N^84^ bonds, respectively.

The deconvolution of the O1s spectra reveals the existence of three peaks. The first peak at 529.8 eV is associated with Fe_2_O_3_/Fe_3_O_4_^19^. The second peak, located at 531.25 eV, corresponds to the Fe–O^85^ bond, which may be attributed to the creation of a bond between the DPH molecule and MS. Furthermore, the third peak at 531.8 eV is assigned to hydrous iron oxide (FeOOH)^86^.

The N1s spectrum presents two peaks at 399 eV and 400.2 eV, which correspond to the N–Fe^86^ and C–N^83^ bonds, respectively. These findings support the theory that the donor/acceptor mechanism is responsible for the establishment of a bond between the N atoms of DPH molecules atoms and Fe metal.

The Fe2p spectrum consists of two main peaks, including Fe2p_3/2_ (~ 710 eV) and Fe2p_1/2_ (~ 724 eV). The deconvolution of the Fe2p_3/2_ spectrum reveals three peaks at binding energies of 709.85 eV, 711.5 eV, and 713.3 eV. The first peak, at 709.85 eV, is related to iron oxides Fe_2_O_3_/Fe_3_O_4_^19^. The second peak at 711.5 eV is ascribed to FeOOH^87^, confirming the O1s and GIXRD spectra results. Additionally, the third peak at 713.3 eV is associated with satellites of Fe (III) and FeCl_3_^87^. The two peaks observed in the Fe2p_1/2_ spectra at 724.4 eV and 726.38 eV linked to Fe^2+^ 2p_1/2_ and Fe^3+^ 2p_1/2_, respectively^54^. The foregoing results demonstrate inhibitor adsorption on the MS surface as a result of the presence of DPH molecule components in the surface film.

### Surface hydrophobicity

**Figure 12:**
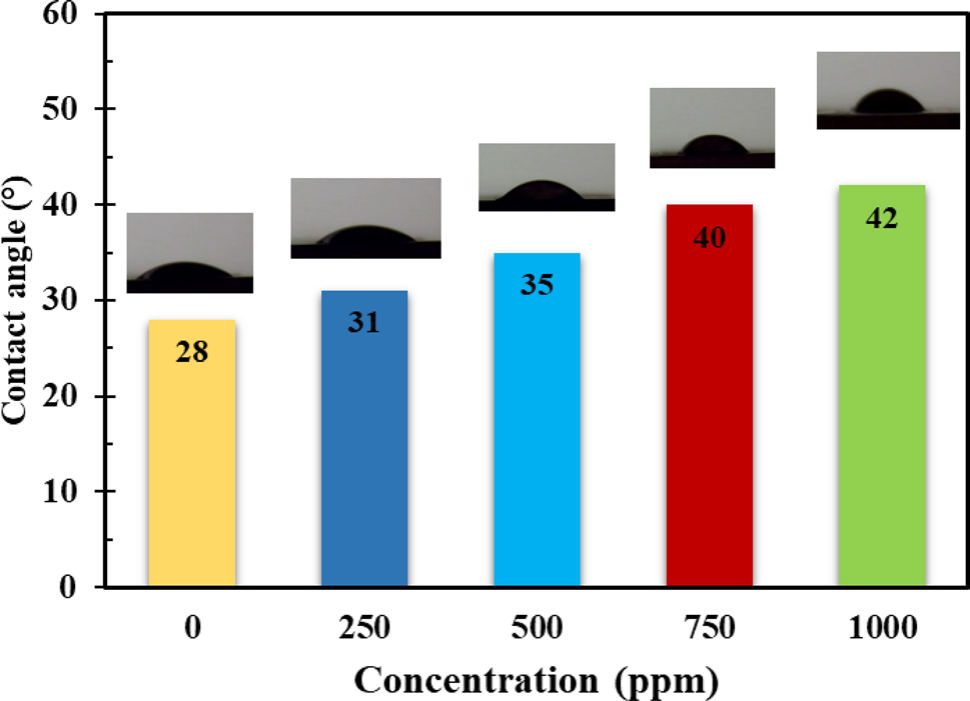
Contact angle of the surface of MS immersed in 1 M HCl solution containing various concentrations of DPH after 4 h.

Contact angle measurement was performed to investigate the effect of the presence of inhibitor on MS surface chemistry. The presented results in Fig. 12 indicate that the inhibitor-free specimen has a low contact angle (28°). This could be due to the development of a hydrogen bond between the iron oxide or hydroxide layers and the corrosive species, which increases surface hydrophilicity^62^. However, when an inhibitor is added, it is noticed that the contact angle rises with increasing DPH concentration, reaching a maximum of 42° at a concentration of 1000 ppm of DPH. DPH molecules adsorbed on the MS surface have increased hydrophobicity because of the existence of non-polar groups such as benzene rings and formation of less hydrophilic corrosion products.

### Computational studies

#### Electronic properties

**Figure 13:**
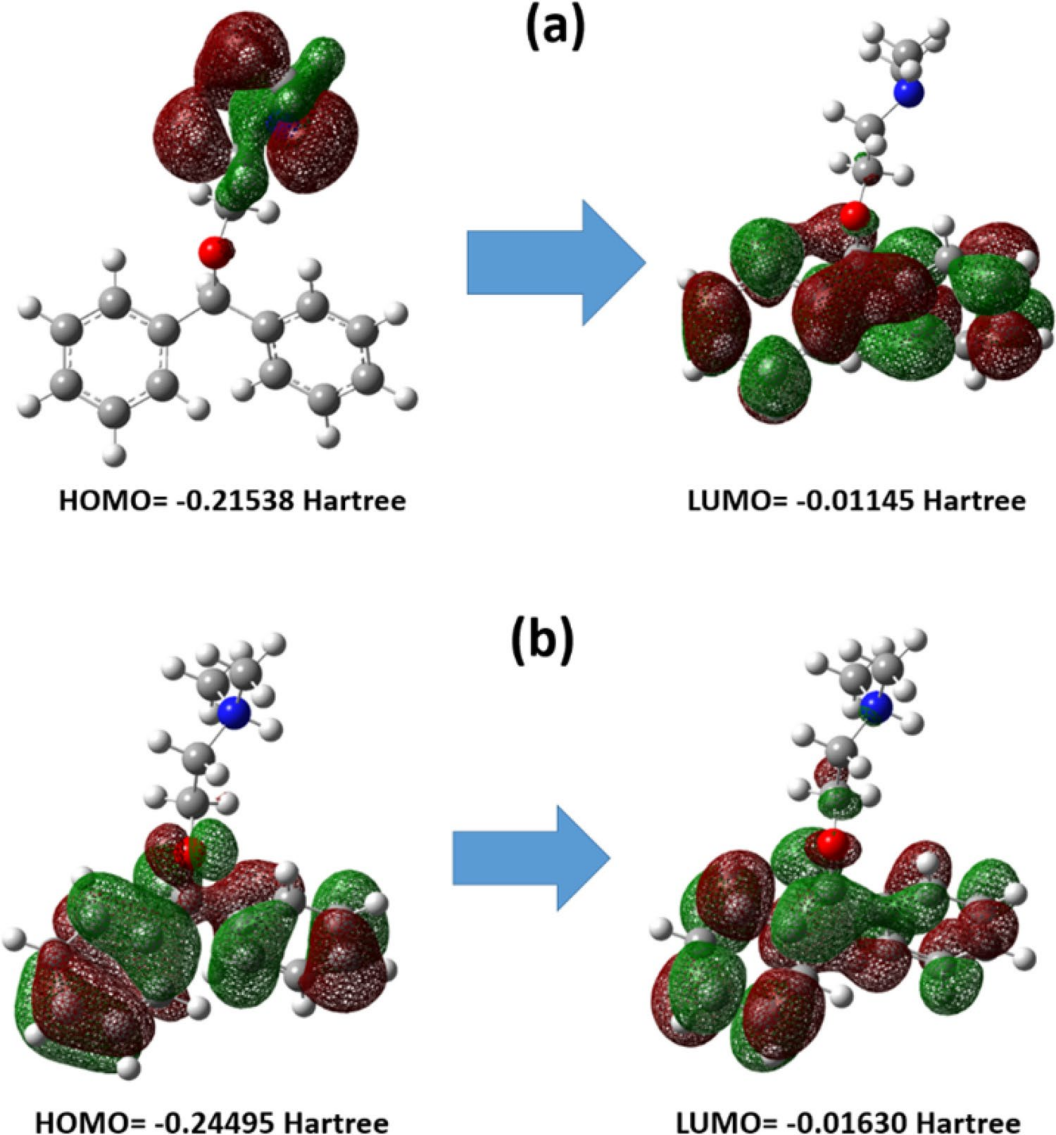
The HOMO and LUMO of neutral (**a**) and protonated (**b**) DPH. The color scheme is the same as Fig. 1. The positive and negative surfaces are presented with red and green colors, respectively.

QM calculations were used to explore the electronic characteristics, as mentioned in section “QM calculations”. Figure 13 depicts the HOMO and LUMO of DPH in neutral and protonated states. The QM parameters of neutral and protonated DPH were computed and are exhibited in Supplementary Information (Table S1). HOMO and LUMO have electrophile and nucleophile properties, respectively. It is revealed that HOMO has a higher eigenvalue than LUMO. A molecule with a small bandgap may be more capable of participating in donor–acceptor interactions.

The DPH partial atomic charges used for Fukui indices computations are reported in Supplementary Information (Table S2). The outcomes for electrophilic and nucleophilic attacks of neutral DPH are also provided in Supplementary Information (Fig. S2). The represented results in Fig. S2a and Table S2 indicate that a considerable value of $f^{ - }$ function on nitrogen atom enables it to give electrons when attacked by an electrophile. As shown in Fig. S2b, the carbon atoms of the aromatic ring have large values of $f^{ + }$ function. Therefore, the DPH nucleophilic attacks are located on these atoms. By comparing Fig. 13 and Fig. S2, it can be concluded that the electrophilic and nucleophilic sites which are recognized with Fukui indices are in accordance with HOMO and LUMO, respectively. The partial charges and partial Fukui indices of protonated DPH are stated in Supplementary Information (Table S3), and also the schematic findings for electrophilic and nucleophilic attacks of protonated DPH are presented in Supplementary Information (Fig. S3). The comparison of the results of Fig. S3a and Table S3 demonstrate that aromatic ring and oxygen atom possess large $f^{ - }$ function values and electrophilic attack. As shown in Fig. S3b, the nucleophilic attacks of protonated DPH are located on the hydrogen atom bonded to the nitrogen atom.

#### Molecular interactions

**Figure 14:**
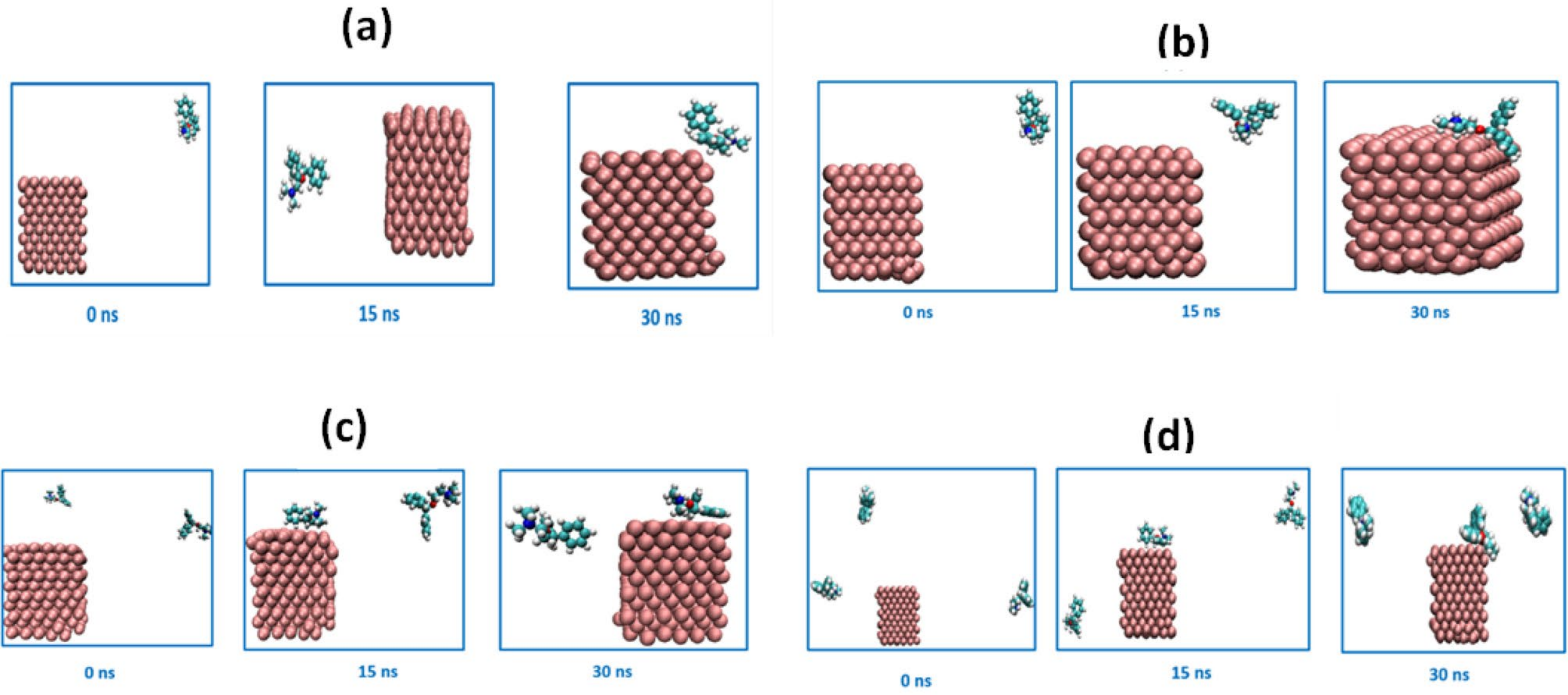
The snapshot of adsorption of DPH on the MS crystal at 250 ppm (**a**), 500 ppm (**b**), 750 ppm (**c**), and 1000 ppm (**d**) concentrations during simulation time. The carbon, nitrogen, hydrogen, oxygen, and iron are shown with cyan, blue, white, red, and purple vdW spheres, respectively. The water and acid molecules were eliminated for the clarity of the plot. The adsorption of DPH molecules on iron at 250 ppm. 500 ppm, 750 ppm, and 1000 ppm concentrations are shown in Videos S1, S2, S3, and S4 in the supplementary information, respectively.

The snapshots of the simulation boxes for different concentrations of DPH are illustrated in Fig. 14. As depicted in this figure, the DPH molecules are first spread haphazardly around the MS crystal. As simulation proceeds, the distance between DPH molecules and MS crystal declines, strengthening the interaction energy between them. Following DPH molecule adsorption on the MS crystal, the interaction potential decreases to a minimum value at equilibrium. Figure 14 depicts the vdW interactions between DPH molecules and MS crystals at various concentrations. The Lennard–Jones (LJ) potential between DPH and MS is proportional to the binding energy between them. The LJ potential (Supplementary Information, Fig. S4) between DPH and MS is weak at 250 ppm concentration. Also, as it is depicted in Fig. 14a at a concentration of 250 ppm, the DPH molecule has not been adsorbed on the MS surface during 30 ns. When the DPH concentration increased from 250 to 500 ppm, the LJ potential increased to 195 kJ/mol. It is demonstrated in Fig. 14b that the DPH molecule has been adsorbed on the MS at 500 ppm, which has higher binding energy than 250 ppm. By increasing concentration from 500 to 750 ppm, the binding energy reached 270 kJ/mol. The comparison of LJ potentials between 750 and 1000 ppm indicates that the increasing concentration in this range does not considerably affect the binding energy between the DPH and MS.

#### Adsorption sites

The radial distribution function analysis was performed to determine the configuration of DPH molecules on the surface of MS crystal. The radial probability of finding MS atoms around the benzene rings, nitrogen, and oxygen atoms of DPH at four concentrations is depicted in Supplementary Information (Fig. S5). The radial probability of MS atoms around the DPH at 250 ppm in Fig. S5a indicates that benzene ring number one (Ring 1) is placed at the distance of 2.16 from the MS (the first peak of the blue curve in Fig. S5a). The benzene ring number 2 (Ring 2), oxygen, and nitrogen atoms of the DPH are at the radius of 2.60 nm from the MS atoms (red, yellow, and violet curves in Fig. S5a). By increasing concentration from 250 to 500 ppm, as seen in Fig. S5b, the distance between Ring 2 and the oxygen atom of DPH reached 0.60 nm. The short distance between the DPH and MS at a concentration of 500 ppm confirms the stronger adsorption and binding energy observed in Fig. 14 and Fig. S4. Inspection of Fig. S5b indicates that the DPH has adsorbed on the MS from the side of Ring 2 and oxygen atom, while Ring 1 and nitrogen atom are placed at longer distances from the MS. The radial distribution of the DPH at the concentrations of 750 ppm and 1000 ppm are depicted in Fig. S5c and Fig. S5d, respectively. The location and configuration of the DPH at concentrations of 750 ppm and 1000 ppm are the same as the concentration of 500 ppm.

#### Hydrophilicity

For investigation of the corrosion inhibition mechanism of the DPH, the changes in hydrophilicity behaviors of the MS after adsorption of the DPH were considered. The LJ potential between MS and water is illustrated in Supplementary Information (Fig. S6). The LJ potential between the MS and DPH is strong (4340.60 kJ/mol) in a blank solution (0 ppm). By increasing the concertation of DPH to 250 ppm and 500 ppm, the LJ potential between the MS and water reached 4327.50 and 4214.10, respectively. The adsorption of DPH on the MS surface has decreased the binding energy between the MS and water molecules. As discussed in Fig. 14 and Fig. S4, due to the robust adsorption of the DPH on the MS surface at 500 ppm, the decrement of LJ potential between MS and water molecules is remarkable in the concentration of 500 ppm. By increasing the concentration of the DPH to 750 ppm and 1000 ppm, the LJ potential between the MS and water molecules reached 4080.50 kJ/mol, which confirms the inhibition mechanisms at 750 ppm and 1000 ppm are almost similar. The radial probability of finding oxygen atoms of water molecules around the MS was calculated and presented in Supplementary Information (Fig. S7). By increasing the concentration of DPH, the height of the peak in Fig. S7 decreased, which confirms the increment of hydrophobic behavior of MS after inhibitor adsorption.

## Conclusion

The inhibition properties of DPH as an MS corrosion inhibitor have been experimentally and theoretically investigated successfully. The results of the electrochemical examination in 6 h showed that when the inhibitor concentration grew from 0 to 750 ppm, the corrosion efficiency improved, and at 1000 ppm, the corrosion efficiency was practically identical to 750 ppm (about 91%). However, after 24 h of immersion, charge transfer resistance decreased at concentrations of 0–750 ppm. DPH adsorption on the MS surface was established by FTIR and UV–Vis analysis, and the existence of N–Fe and O–Fe bonds in XPS analysis verified the aforesaid findings. So, the creation of a dense layer on the MS surface as a result of DPH physical and chemical adsorption on the metal surface lowered the corrosion rate. The MD simulation and QM calculations showed that DPH molecules strongly adsorbed on the MS by increasing concentration from 250 to 500 ppm and 750 ppm. In comparison, the increment of concertation from 750 to 1000 ppm does not significantly affect the binding energy. The radial distribution analysis indicates that the DPH has adsorbed on the MS at a distance of 2.5 nm, while this distance decreased to 0.6 nm in higher concentrations of DPH inhibitor. The simulation results showed that the adsorption of DPH on the MS has decreased the binding energy between the MS and water molecules, confirming experimental results. Finally, the DPH molecules orientation on the surface was assessed to be horizontal on the MS surface based on electrochemical data, which was confirmed by computational studies.

## Supplementary Information

Supplementary Information.

Supplementary Video S1.

Supplementary Video S2.

Supplementary Video S3.

Supplementary Video S4.

## Data Availability

The datasets used and/or analysed during the current study available from the corresponding author (Mohammad Mahdavian).
