# Supplementary material for: Corrosion inhibition of a novel antihistamine-based compound for mild steel in hydrochloric acid solution: experimental and computational studies
Source: Sci Rep. 2022 Aug 4;12:13450. doi: 10.1038/s41598-022-17589-y (PMC9352695; doi:10.1038/s41598-022-17589-y)
Supplement: Supplementary file 1 — Supplementary Information. [file 41598_2022_17589_MOESM1_ESM.docx]

**Supplementary Information for**

**Corrosion inhibition of a novel antihistamine-based compound for mild steel in hydrochloric acid solution: experimental and computational studies**

*Mohammad Ghaderi^1^, Ahmad Ramazani S.A^1,*^, Azadeh Kordzadeh^1^, Mohammad Mahdavian^2,*^, Eiman Alibakhshi^2^, Arash Ghaderi^3^*

*^1^ Department of Chemical & Petroleum Engineering, Sharif University of Technology, Tehran, Iran*

*^2^ Surface Coating and Corrosion Department, Institute for Color Science and Technology, Tehran, Iran*

*^3^ Department of Chemistry, College of Sciences, University of Hormozgan, Bandar Abbas 7916193145, Iran*

*Corresponding authors:*

*Ahmad Ramazani S.A. ( Email: ramazani@sharif.edu ) & Mohammad Mahdavian ( Email: mahdavian-m@icrc.ac.ir )*


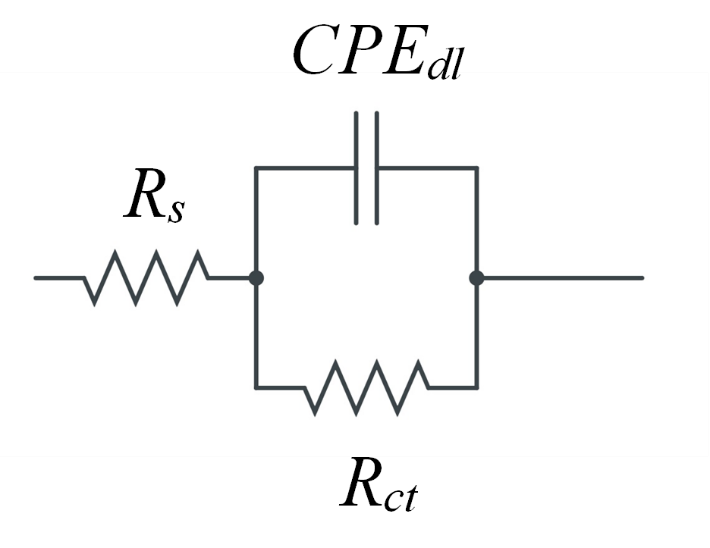


**Figure S1**. Schematic of R(QR) equivalent electrical circuit


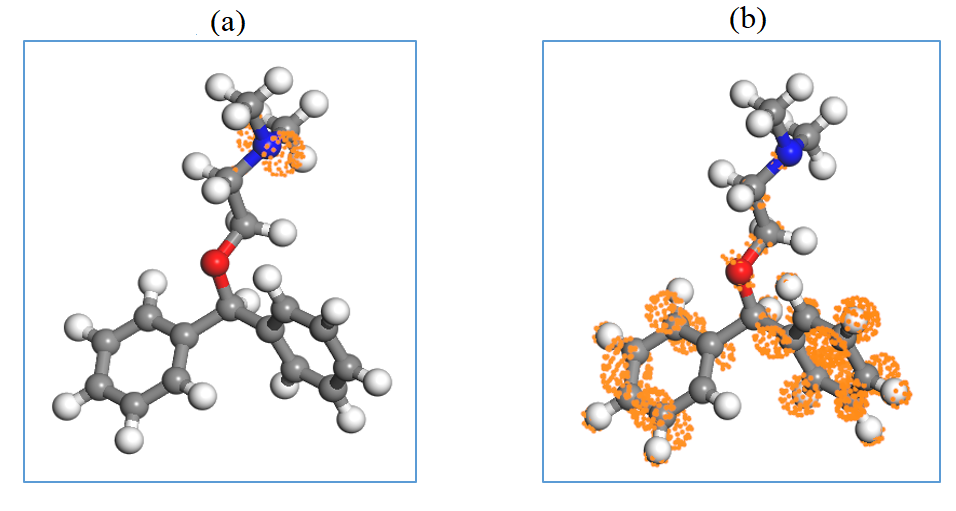


**Figure S2.** The Fukui indices of DPH for (a) electrophilic attack and (b) nucleophilic attack. The carbon, nitrogen, hydrogen, and oxygen are shown with cyan, blue, white, and red spheres, respectively and the Fukui indices are shown with orange color.


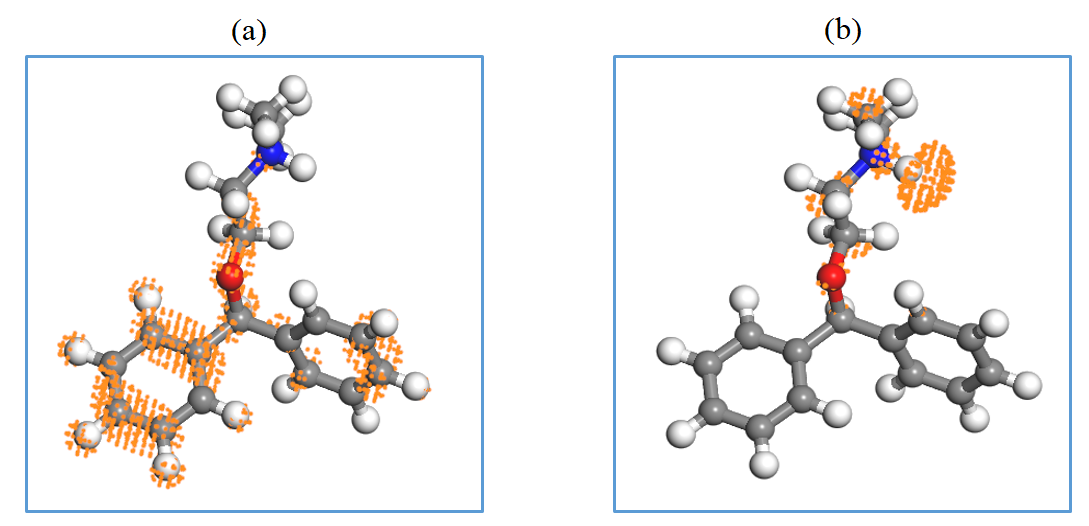


**Figure S3.** The Fukui indices of protonated DPH for (a) electrophilic attack and (b) nucleophilic attack. The color scheme is the same as Fig. S1, and the Fukui indices are shown with orange color.


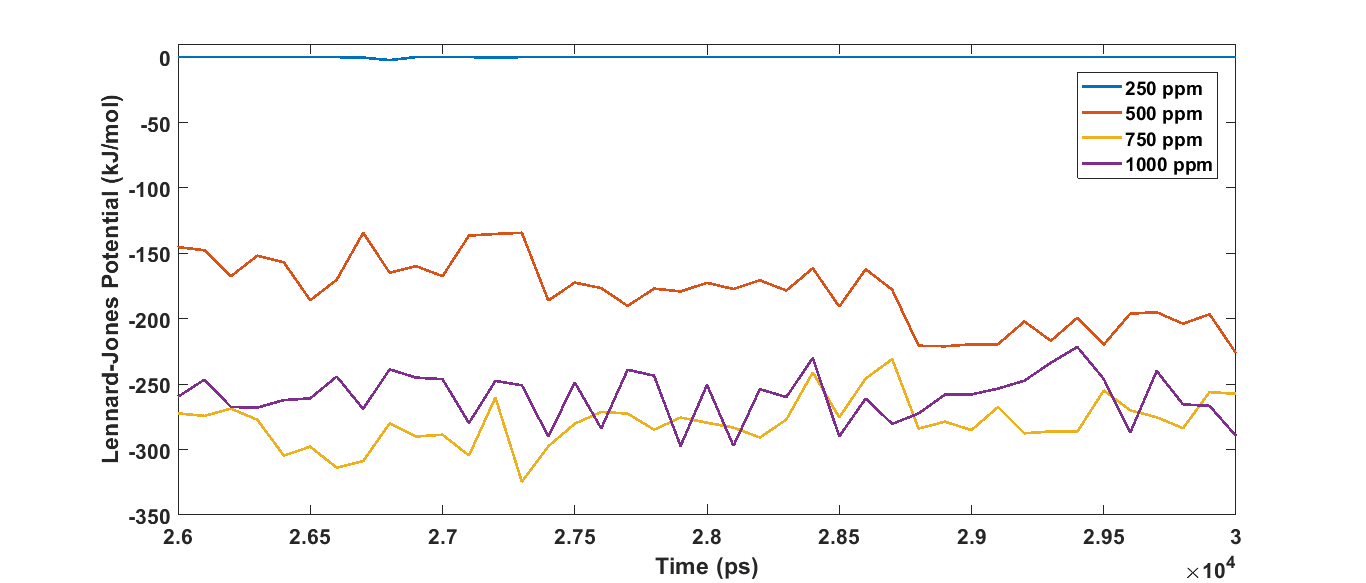


**Figure S4**. Lennard-Jones (LJ) potential energy profile for interaction between DPH molecules and MS at different concentrations.


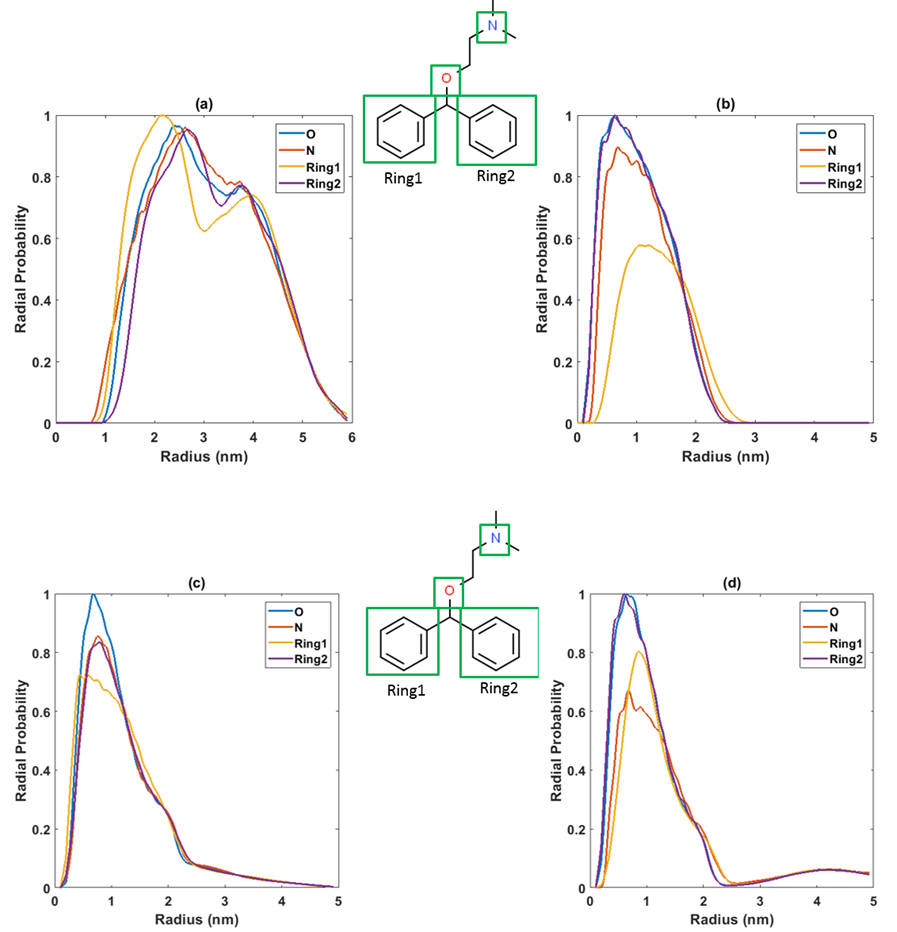


**Figure S5**. The radial probability of iron atoms referenced benzene rings, nitrogen, and oxygen atoms of DPH at concentrations of a) 250 ppm, b) 500 ppm, c) 750 ppm, and d) 1000 ppm.


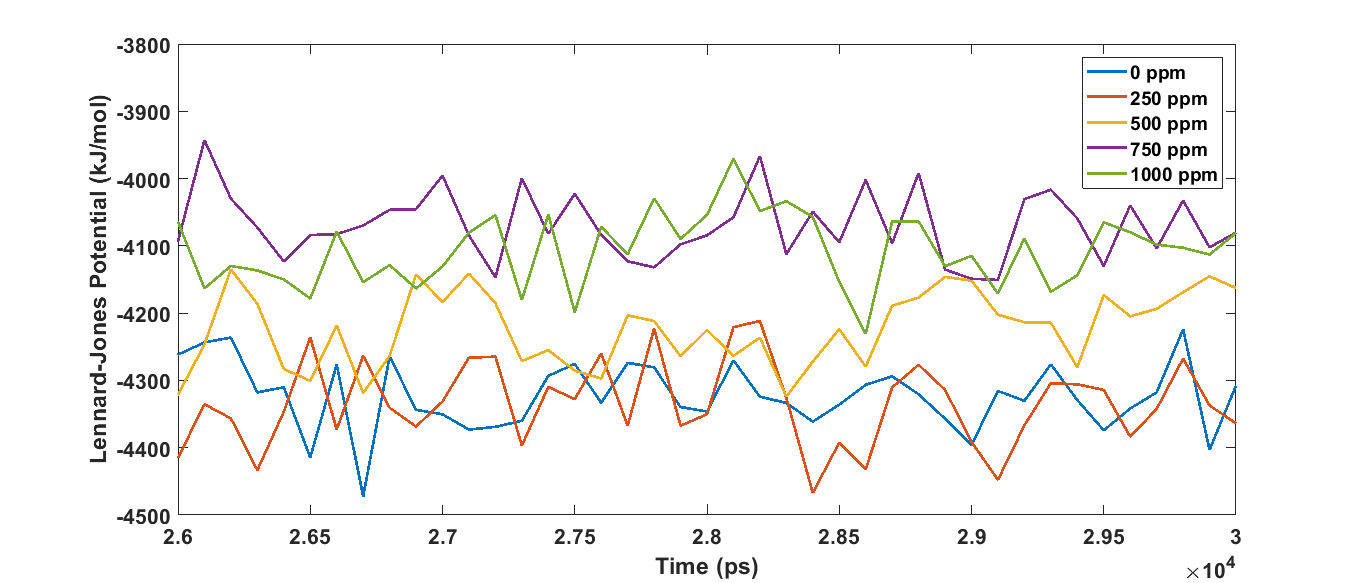


**Figure S6**. Lennard-Jones (LJ) potential energy profile for interaction between water molecules and iron at different concentrations of DPH.


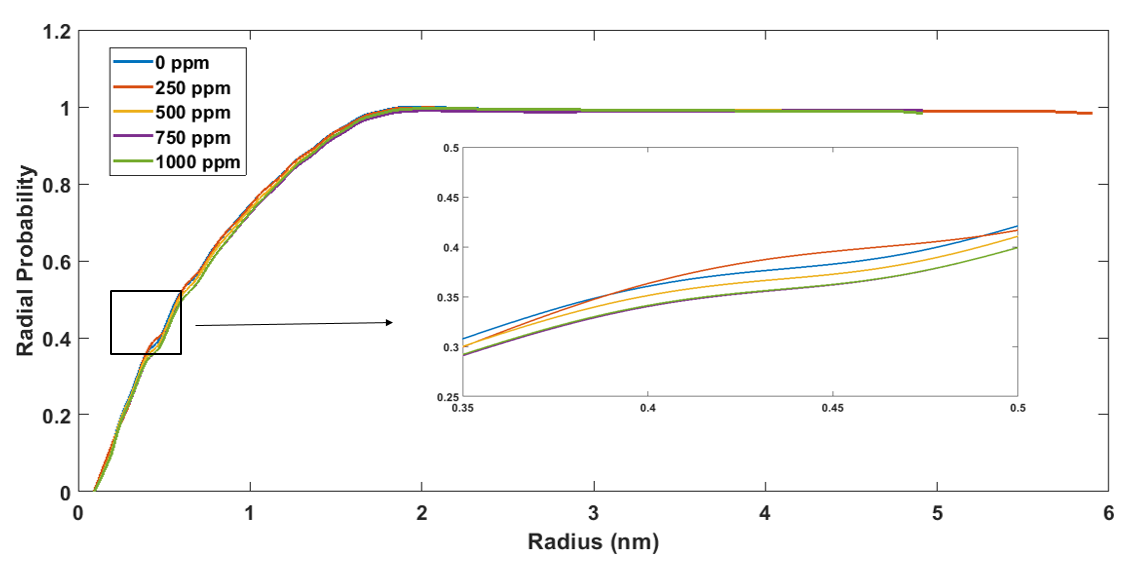


**Figure S7**. The radial probability of oxygen atoms of water molecules referenced the iron crystal.

**Table S1.** The energies (Hartree) of HOMO, LUMO, bandgap *(*$\Delta E_{L-H}$), electron affinity (*A*), Ionization potential (*I*), electronegativity ($\chi$), hardness ($\eta$), fraction of electron transferred ($\Delta N$), polarizabilty (Bohr), and dipole moment (Debye) for neutral (N) and protonated (P) DPH.

|  | N | P |
| --- | --- | --- |
| $\boldsymbol{E}_{\boldsymbol{HOMO}}$ | -0.21538 | -0.24495 |
| $\boldsymbol{E}_{\boldsymbol{LUMO}}$ | -0.01145 | -0.01630 |
| $\boldsymbol{\Delta}\boldsymbol{E}_{\boldsymbol{L-H}}$ | 0.20393 | 0.22865 |
| *I* | 0.21538 | 0.24495 |
| *A* | 0.01145 | 0.01630 |
| $\boldsymbol{X}$ | 0.11341 | 0.13062 |
| $\boldsymbol{H}$ | 0.10196 | 0.11432 |
| $\boldsymbol{\Delta N}$ | 0.25478 | 0.15196 |
| *D* | 1.92590 | 19.60790 |
| $\boldsymbol{a}_{\boldsymbol{xx}}$ | 277.5550 | 250.6140 |
| $\boldsymbol{a}_{\boldsymbol{yy}}$ | 288.6090 | 281.1780 |
| $\boldsymbol{a}_{\boldsymbol{zz}}$ | 150.6860 | 167.5560 |
| Exact polarizability | 716.8500 | 699.34800 |

**Table S2.** The atomic partial charges and Fukui indices for electrophilic, nucleophilic, and radical attacks $\times$ (1.609 $\times{10}^{-19} Coulomb$) of neutral DPH. The atom numbers are the same as **Fig. 1**.

| $f^{0}$ | $f^{+}$ | $f^{-}$ | Charge | Atom | Number |
| --- | --- | --- | --- | --- | --- |
| 0.010 | 0.006 | 0.015 | -0.540 | O | 1 |
| 0.125 | -0.002 | 0.251 | -0.437 | N | 2 |
| 0.008 | 0.011 | 0.005 | 0.078 | C | 3 |
| 0.017 | 0.039 | -0.006 | 0.078 | C | 4 |
| 0.010 | 0.022 | -0.001 | 0.097 | C | 5 |
| 0.005 | 0.003 | 0.007 | 0.056 | C | 6 |
| 0.018 | 0.001 | 0.035 | -0.049 | C | 7 |
| 0.011 | 0.033 | -0.011 | -0.134 | C | 8 |
| 0.015 | 0.023 | 0.008 | -0.141 | C | 9 |
| 0.039 | 0.071 | 0.007 | -0.135 | C | 10 |
| 0.033 | 0.066 | 0.001 | -0.123 | C | 11 |
| 0.024 | 0.002 | 0.047 | -0.171 | C | 12 |
| 0.025 | 0.004 | 0.046 | -0.165 | C | 13 |
| 0.043 | 0.081 | 0.005 | -0.101 | C | 14 |
| 0.043 | 0.068 | 0.017 | -0.105 | C | 15 |
| 0.028 | 0.042 | 0.014 | -0.103 | C | 16 |
| 0.028 | 0.046 | 0.010 | -0.101 | C | 17 |
| 0.037 | 0.059 | 0.015 | -0.098 | C | 18 |
| 0.035 | 0.047 | 0.022 | -0.097 | C | 19 |
| 0.018 | 0.029 | 0.006 | 0.128 | H | 20 |
| 0.012 | 0.009 | 0.015 | 0.108 | H | 21 |
| 0.006 | 0.002 | 0.010 | 0.103 | H | 22 |
| 0.037 | 0.006 | 0.069 | 0.086 | H | 23 |
| 0.018 | 0.001 | 0.035 | 0.118 | H | 24 |
| 0.006 | 0.023 | -0.011 | 0.105 | H | 25 |
| 0.010 | 0.015 | 0.006 | 0.100 | H | 26 |
| 0.020 | 0.034 | 0.007 | 0.103 | H | 27 |
| 0.014 | 0.032 | -0.004 | 0.099 | H | 28 |
| 0.020 | -0.002 | 0.042 | 0.114 | H | 29 |
| 0.025 | 0.009 | 0.041 | 0.114 | H | 30 |
| 0.040 | 0.006 | 0.075 | 0.088 | H | 31 |
| 0.022 | 0.002 | 0.042 | 0.112 | H | 32 |
| 0.041 | 0.007 | 0.074 | 0.088 | H | 33 |
| 0.026 | 0.008 | 0.043 | 0.112 | H | 34 |
| 0.023 | 0.041 | 0.005 | 0.102 | H | 35 |
| 0.023 | 0.035 | 0.012 | 0.102 | H | 36 |
| 0.021 | 0.029 | 0.012 | 0.103 | H | 37 |
| 0.019 | 0.030 | 0.009 | 0.103 | H | 38 |
| 0.023 | 0.035 | 0.012 | 0.100 | H | 39 |
| 0.022 | 0.030 | 0.014 | 0.100 | H | 40 |

**Table S3.** The atomic partial charges and Fukui indices for electrophilic, nucleophilic, and radical attacks $\times$ (1.609 $\times{10}^{-19}\mathrm{Coulomb}$) of protonated DPH. The atom numbers are the same as **Fig. 1**.

| $f^{0}$ | $f^{+}$ | *f^−^* | Charge | Atom | Number |
| --- | --- | --- | --- | --- | --- |
| 0.012 | 0.011 | 0.012 | -0.528 | O | 1 |
| 0.032 | 0.064 | 0.001 | -0.445 | N | 2 |
| 0.007 | 0.002 | 0.012 | 0.068 | C | 3 |
| 0.004 | -0.005 | 0.013 | 0.063 | C | 4 |
| 0.041 | -0.008 | 0.090 | 0.105 | C | 5 |
| 0.009 | 0.012 | 0.006 | 0.052 | C | 6 |
| 0.016 | 0.031 | 0.002 | -0.095 | C | 7 |
| 0.001 | -0.014 | 0.016 | -0.127 | C | 8 |
| 0.020 | 0.004 | 0.035 | -0.133 | C | 9 |
| 0.015 | 0.008 | 0.022 | -0.124 | C | 10 |
| 0.037 | -0.004 | 0.079 | -0.120 | C | 11 |
| 0.028 | 0.053 | 0.003 | -0.205 | C | 12 |
| 0.031 | 0.057 | 0.005 | -0.200 | C | 13 |
| 0.020 | 0.000 | 0.040 | -0.100 | C | 14 |
| 0.047 | 0.011 | 0.083 | -0.103 | C | 15 |
| 0.019 | 0.016 | 0.022 | -0.101 | C | 16 |
| 0.026 | 0.008 | 0.044 | -0.102 | C | 17 |
| 0.031 | 0.013 | 0.049 | -0.096 | C | 18 |
| 0.063 | 0.015 | 0.110 | -0.094 | C | 19 |
| 0.016 | 0.003 | 0.030 | 0.127 | H | 20 |
| 0.014 | 0.022 | 0.007 | 0.142 | H | 21 |
| 0.015 | 0.021 | 0.008 | 0.123 | H | 22 |
| 0.020 | 0.034 | 0.005 | 0.190 | H | 23 |
| 0.022 | 0.042 | 0.003 | 0.187 | H | 24 |
| 0.005 | -0.005 | 0.014 | 0.102 | H | 25 |
| 0.015 | 0.003 | 0.026 | 0.102 | H | 26 |
| 0.009 | 0.008 | 0.010 | 0.103 | H | 27 |
| 0.016 | -0.005 | 0.038 | 0.099 | H | 28 |
| 0.020 | 0.041 | -0.001 | 0.184 | H | 29 |
| 0.031 | 0.053 | 0.008 | 0.185 | H | 30 |
| 0.023 | 0.042 | 0.004 | 0.190 | H | 31 |
| 0.028 | 0.054 | 0.002 | 0.183 | H | 32 |
| 0.024 | 0.043 | 0.005 | 0.189 | H | 33 |
| 0.030 | 0.052 | 0.008 | 0.183 | H | 34 |
| 0.013 | 0.002 | 0.024 | 0.103 | H | 35 |
| 0.025 | 0.009 | 0.041 | 0.103 | H | 36 |
| 0.016 | 0.012 | 0.020 | 0.105 | H | 37 |
| 0.021 | 0.007 | 0.035 | 0.104 | H | 38 |
| 0.018 | 0.011 | 0.026 | 0.102 | H | 39 |
| 0.028 | 0.011 | 0.046 | 0.102 | H | 40 |
| 0.133 | 0.266 | 0.001 | 0.372 | H | 41 |
